# Risk of death in England following a positive SARS-CoV-2 test: A retrospective national cohort study (March 2020 to September 2022)

**DOI:** 10.1371/journal.pone.0304110

**Published:** 2024-10-09

**Authors:** Clarissa Bauer-Staeb, Richard James Holleyman, Sharmani Barnard, Andrew Hughes, Samantha Dunn, Sebastian Fox, Justine Fitzpatrick, John Newton, Paul Fryers, Paul Burton, Peter Goldblatt

**Affiliations:** 1 Office for Health Improvement and Disparities, London, United Kingdom; 2 UK Health Security Agency, London, United Kingdom; 3 Population Health Sciences Institute, Newcastle University, Newcastle upon Tyne, United Kingdom; 4 School of Population Health, Curtin University, Bentley, Western Australia, Australia; 5 Department of Epidemiology & Public Health, UCL Institute of Health Equity, University College London, London, United Kingdom; Gabriele d’Annunzio University of Chieti and Pescara: Universita degli Studi Gabriele d’Annunzio Chieti Pescara, ITALY

## Abstract

**Background:**

We aimed to estimate the relative risk of mortality following a first positive SARS-CoV-2 test during the first, second, and third waves of the COVID-19pandemic in England by age, sex, and vaccination status, taking into account pre-existing health conditions and lifestyle factors.

**Methods:**

We conducted a retrospective cohort study of all individuals registered with the National Health Service (NHS) in England from 1 March 2020 to September 2022. Data for all individuals were obtained and linked including primary care records, hospital admission episodes, SARS-CoV-2 test results, vaccinations, and death registrations. We fitted Cox Proportional Hazards models with time dependent covariates for confirmed SARS-CoV-2 infection to model the risk of subsequent mortality.

**Results:**

The hazard ratio for death after testing positive for subsequent, compared with those not testing positive, amongst unvaccinated individuals, ranged from 11 to 89 by age and sex, in the first four weeks following a positive test in wave one and reduced to 14 to 50 in wave three. This hazard was further reduced amongst those who had three vaccines to between 1.4 and 7 in wave three.

**Conclusions:**

This study provides robust estimates of increased mortality risk among those who tested positive over the first three waves of the COVID-19 pandemic in England. The estimates show the impact of various factors affecting the risk of mortality from COVID-19. The results provide the first step towards estimating the magnitude and pattern of mortality displacement due to COVID-19, which is essential to understanding subsequent mortality rates in England.

## Plain language summary

The COVID-19 pandemic caused many deaths over a prolonged period in England, substantially increasing population mortality rates at the time, disrupting mortality trends, and displacing forward many deaths that would have been expected in subsequent years. Research to date has described greater mortality in those with pre-existing health conditions or who were otherwise more clinically vulnerable. However, such research has generally been limited to samples of the population, mortality in specific subgroups, or during certain “waves” of the pandemic. Population level statistics have been published on excess mortality throughout the pandemic by cause. These are however based on recording of COVID-19 on the death certificate. There is a need to study whole populations to directly assess mortality risk following a positive test for SARS-CoV-2 and how it varies in different groups, and to assess the likely impact of COVID-19 mortality that occurred during the pandemic period on future mortality rates.

The study uses data on the whole population to assess the relative risk of death following a positive SARS-CoV-2 test result in England and how it varies by age, sex, and vaccination status over the first three “waves” of the pandemic, as well as providing relative mortality ratios by socio-demographic status and geography, comorbidities, and clinical vulnerability. These risk estimates have then been combined to approximate the overall mortality risk from COVID-19 compared with the pre-existing risk for groups of individuals with specific characteristics. Thus, allowing us to assess the likely impact of COVID-19 on future mortality trends due to mortality displacement by the COVID-19 pandemic.

Understanding patterns of mortality risk following confirmed SARS-CoV-2 infection is important for understanding the impact of the pandemic. Documenting variation in mortality rates between specific population groups enables planning for any future pandemic but is also desirable in reflecting on the experience and handling of the current pandemic. Many individuals died earlier than would otherwise have been the case because of COVID-19. At a population level, this resulted in excess deaths during the height of the pandemic but also fewer deaths than expected during some later periods. This analysis provides a basis for estimating the likely effect of this mortality displacement on future mortality rates.

## Introduction

The COVID-19 pandemic caused prolonged periods of excess mortality in many countries. In England, between March 2020 and February 2023, there were 154,030 deaths in excess of what might have been expected had the pandemic not occurred [1]. The risk of mortality following COVID-19 is known to differ between individuals, with various factors impacting the risk including, but not limited to, sex, ethnicity, deprivation, and certain health conditions [2–6]. Risk of death also varied between waves of the pandemic, due to changing virulence of the dominant viral strain and the impact of counter measures such as shielding for those at greatest risk, specific treatments for diagnosed cases, and vaccine protection [2, 3, 7].

Existing studies assessing risk of mortality from COVID-19 in relation to comorbidities were important in informing shielding, vaccination, and therapeutic programmes and other public health measures [3, 8, 9]. However, these studies have been based on samples of the population, examined the impact of COVID-19 in specific population subgroups, or have only explored specific waves of the pandemic. There is a need for a more complete assessment of mortality risk based on a national population.

We quantify the excess risk of death following confirmed SARS-CoV-2 infection by age and sex using a national population cohort of nearly 63 million individuals in England registered with the NHS for whom we have linked data from primary care records, hospital admissions, SARS-CoV-2 infection test results, vaccination status, and mortality. The risk of death after infection could also be estimated according to socio-demographic characteristics, presence of other morbidities, and vaccine status during successive waves of COVID-19.

One reason for wanting to document mortality risk during the pandemic more precisely and with more precision is because those who died from COVID-19 will not contribute to subsequent mortality rates. To estimate the extent to which the pandemic will influence future mortality trends through mortality displacement, it is necessary to understand in detail the pre-existing risk of the affected population groups and how it increased following COVID-19 [10]. This work is necessary to estimate likely future mortality rates following the pandemic [11].

## Methods

### Study design & data sources

We conducted a retrospective cohort of the national population of all individuals registered with the NHS in England from 1 March 2020, with SARS-CoV-2 cases being followed until 31 March 2022 and mortality until 2 September 2022. All individuals registered as alive within NHS England’s National Immunisation Management System (NIMS) database were extracted along with personal identifiable information. The NIMS dataset includes individuals registered with the National Health Service (NHS) who are alive in the resident population, along with further information regarding COVID-19 vaccination history, ethnic group, postcode of residence, and indicator flags for individuals identified as ‘clinically extremely vulnerable’ (CEV). This individual-level dataset was linked deterministically using a match rank process with a combination of NHS number, date of birth, sex and residence postcode (see S1 Appendix) to the National SARS-CoV-2 testing data [12], Hospital Episode Statistics (HES) [13], General Practice Extraction Service (GPES) [14], and Office for National Statistics (ONS) mortality data in order to derive socio-demographic and health characteristics and define mortality endpoints. Further details of the linkage methodology and dataset preparation are provided in the S1 Appendix.

Of the 62,564,742 registered individuals who were born before the 1 March 2020 and registered as alive, those with missing or invalid age and/or sex (n = 185,886, 0.3%) and where no match could be found in GPES (n = 2, <0.01%) were excluded. Furthermore, we excluded those who had a SARS-CoV-2test prior to 1 March 2020, SARS-CoV-2 tests that were conducted post-mortem, and those who had a vaccination date prior to 1 March 2020 (n = 4593, <0.01%). All individuals who had a positive COVID-19 test that occurred after 31 March 2022, after which widespread community testing stopped, were censored at the week prior to a positive test.

### Main exposure

For all individuals, we identified the date they first tested positive for SARS-CoV-2. We did not consider subsequent positive SARS-CoV-2 tests as part of our main exposure, whether these occurred during the initial infection or associated with any later re-infections.

### Primary outcome

Date of death (any cause) was captured for all individuals from ONS mortality data and used to derive survival time in weeks from 1 March 2020.

### Socio-demographic, health characteristics, and vaccination status

Age was defined for each individual on 1 March 2020, using date of birth. Ethnicity was captured from the NIMS dataset and supplemented through linkage to GPES (see S1 Appendix for details) [14, 15]. Social deprivation was measured using the 2019 Index of Multiple Deprivation (IMD), a measure of area deprivation based on ONS Lower-layer Super Output Areas (LSOA) attributed ecologically to individuals [16]. All 32,844 English LSOAs were ranked from most to least deprived and categorised into quintiles. The date of administration of every SARS-CoV-2 vaccination (including 1st, 2nd and booster vaccine) was captured for all individuals.

Health characteristics and co-morbidities were defined for each individual as at 1 March 2020, using disease specific International Classification of Diseases 10 (ICD-10) and SNOMED clinical terminology (SNOMED-CT) codes identified from eligible HES (including any hospital admissions captured by the time of receipt of data) and GPES records respectively [17, 18]. These comprised both physical and mental health diagnoses captured in primary and secondary care including: history of asthma, atrial fibrillation, cancer, heart failure, palliative care, cardiovascular disease, diabetes, chronic kidney disease, dementia, coronary heart disease, learning difficulties, stroke and transient ischaemic attack, liver cirrhosis, epilepsy, bipolar disorder and schizophrenia. Smoking was also included as a behavioural risk factor. Diagnosis codes used to categorise each diagnosis are detailed in S1 Appendix and the supplementary data dictionary (S4 Appendix). We did not use ICD-10 codes captured from mortality records to infer any health characteristics of subjects in our study.

### Statistical analysis

We utilised Cox Proportional Hazard models, using a calendar time baseline hazard function, to quantify the hazard ratio of a positive SARS-CoV-2 test adjusted for sociodemographic, clinical, and health characteristics for each 5-year age group and sex strata. We use the term relative risk synonymously with hazard ratio for clarity and brevity in the manuscript. To measure the effect of SARS-CoV-2, the model included time-dependent terms. Dummy variables were created to denote SARS-CoV-2 positivity in two discrete time periods after a positive test (0 to 4 weeks and 5 to 26 weeks); waves in the pandemic were chosen pragmatically according to the number of deaths recorded during the indicated periods (rather than based upon any predominant variants at the time) and approximated by the periods March 2020 to September 2020, October 2020 to March 2021, and April 2021 onwards and vaccination status at the time of a positive test was based on the number of vaccinations (0 to 3). Constructing variables in this way enabled us to quantify how the effect on mortality of confirmed SARS-CoV-2 varied by period after infection, wave, and vaccination status. We restricted the protective effect of vaccination in our model only to cases with a positive test. In order to obtain the overall risk of death for a particular group of individuals at a given point in time, the baseline hazard is multiplied by the risks associated with all covariates that take values other than zero. The reference category is those where the covariates take a value of zero.

Further details concerning the specification of this model are described in S2 Appendix. We attempted to fit models to all age groups, but convergence was problematic in age groups under 45 due to a low number of deaths. We were therefore unable to model reliable point estimates for these younger age groups and present results for 45 and above by 5-year age groups. Baseline hazards were extracted from the Cox models to determine any consistency of pattern over time and within age groups.

### Study approvals

Public Health England (PHE) had legal permission, provided by Regulation 3 of The Health Service (Control of Patient Information) Regulations 2002, to process confidential patient information without consent under Sections 3 as part of its responsibility to manage the COVID-19 pandemic response [18]. As such, this study, which was undertaken to inform that response, falls outside the remit for ethical review.

## Results

The registered population alive in England on 1 March 2020 included 62,374,261 individuals. We present detailed results for an exemplar age group–those aged between 65 and 69 –and summarise results across the broader age groups. Detailed accounts of the descriptive characteristics, the baseline hazard, and the hazard ratios derived from the models across all age groups are presented in S1–S3 Figs and S1, S2 Tables. The primary focus is on the risk of death associated with COVID-19; however, a commentary on the impact of sociodemographic and health characteristics on risk of death can be found in S3 Appendix.

Among the 65–69 age group, the study population included 1,274,145 females who did not have a positive SARS-CoV-2 test and 197,251 females who had a positive test (Table 1). There were 1,213,395 males who did not have a positive SARS-CoV-2 and 199,829 who tested positive. Vaccine uptake was high amongst this age group with approximately 85–89% having two vaccinations and a booster. Considering mortality across the entire period of follow-up, amongst those who never recorded a positive test for SARS-CoV-2, there were a higher proportion of deaths amongst males (3.8%) than females (2.5%). There were more deaths amongst those who tested positive for SARS-CoV-2, and reflecting the same gender pattern (5.3% and 3.5% for males and females, respectively).

**Table 1:** Vaccination and survival characteristics by gender and positive SARS-CoV-2 test status for those aged 65–69 (as an exemplar group).

	65-69-year-olds			
	Female		Male	
	No positive SARS-CoV-2 test	Positive SARS-CoV-2 test	No positive SARS-CoV-2 test	Positive SARS-CoV-2 test
n	1,274,145	197,251	1,213,395	199,829
1st Vaccination	1,178,744 (92·5)	188,169 (95·4)	1,100,381 (90·7)	189,661 (94·9)
2nd Vaccination	1,165,584 (91·5)	186,411 (94·5)	1,087,335 (89·6)	187,986 (94·1)
Booster Vaccination	1,104,652 (86·7)	175,857 (89·2)	1,030,480 (84·9)	178,556 (89·4)
Deaths*	31,781 (2·5)	6,809 (3·5)	45,926 (3·8)	10,508 (5·3)
Survival times (median [IQR])	130 [130·0, 130·00]	130 [130·0, 130·00]	130 [130·0, 130·00]	130 [130·0, 130·00]

Data are presented as n (%) unless otherwise specified. 1st positive COVID-19 tests from 1 March 2020 to 31 March 2022. *Deaths are defined according to censoring.

### Mortality risk

We derived the baseline hazards from the Cox Proportional Models–these showed a consistent pattern across age groups for each sex (see S1 Fig). All models were adjusted for a range of sociodemographic, clinical and health characteristics with the relative risks for each 5-year age group and sex strata presented in detail S2, S3 Figs and S2 Table. There was a distinct initial peak at the beginning of the pandemic, when there was limited community SARS-CoV-2 testing, and a similar (smaller) peak at the time of the second wave (winter 2020–21).

### Risk of 1st positive SARS-CoV-2 test for ages 65–69

**Fig 1:**
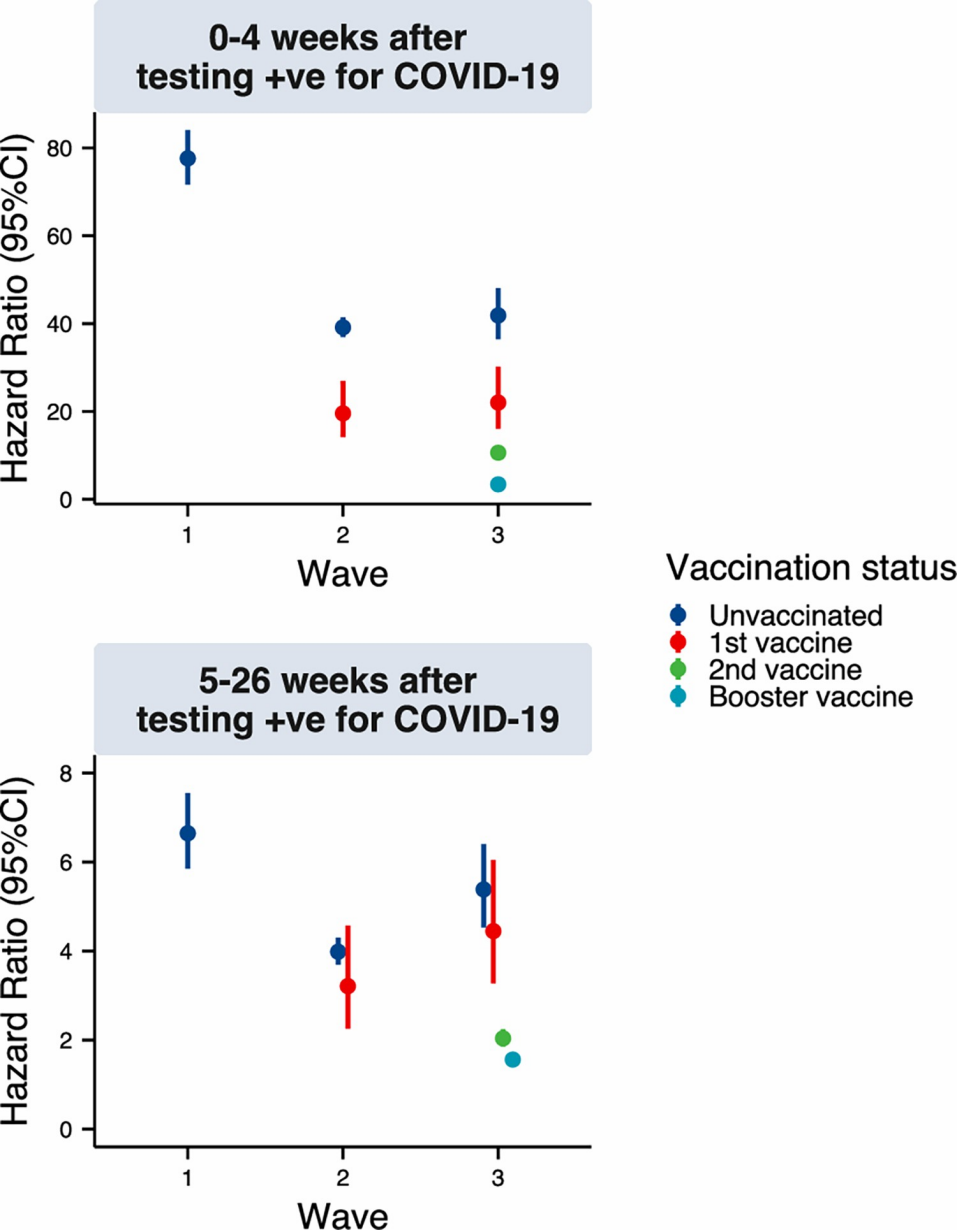
Hazard ratios (and 95% confidence intervals) for the effect of testing positive for COVID-19 on mortality by time-period of infection and vaccination status.

For both sexes in our exemplar age group, the first four weeks following a first positive SARS-CoV-2 test represented the greatest increase in risk among all modelled risk factors (Fig 1 and Table 2). The relative risk of mortality associated with a positive test then decreased with time after infection (Fig 1 and Table 2); for unvaccinated females during wave 1, the relative risk was 77.62 in weeks 0 to 4 and 6.65 in weeks 5 to 26. The relative risks also decreased with increased numbers of vaccinations and in subsequent waves of the pandemic. For example, during wave three the immediate relative risk in weeks 0 to 4 among unvaccinated females was 41.87 (95% CI = 36.48 to 48.05), amongst those who had received one vaccine the hazard was 22.03 (95% CI = 16.08 to 30.17), with two vaccines it was 10.61 (95% CI = 9.62 to 11.70) and with a 3rd vaccine it was 3.41 (95% CI = 2.99 to 3.89).

**Table 2:** Hazard ratios (and 95% confidence intervals) derived from the Cox Proportional Hazard model denoting the hazard of deaths in those aged 65 to 69 stratified by sex.

	65-69-year-olds	
	Female	Male
**Wave & COVID-19 & Vaccination status**		
Wave 1 & 0–4 weeks & unvaccinated	77·62 (71·67 to 84·07)	79·21 (74·62 to 84·07)
Wave 1 & 5–26 weeks & unvaccinated	6·65 (5·85 to 7·55)	6·01 (5·42 to 6·68)
Wave 2 & 0–4 weeks & unvaccinated	39·15 (36·97 to 41·45)	42·16 (40·29 to 44·12)
Wave 2 & 0–4 weeks & 1st vaccine	19·57 (14·2 to 26·96)	22·06 (17·02 to 28·6)
Wave 2 & 5–26 weeks & unvaccinated	3·99 (3·69 to 4·3)	4·34 (4·08 to 4·61)
Wave 2 & 5–26 weeks & 1st vaccine	3·21 (2·26 to 4·57)	3·02 (2·2 to 4·13)
Wave 3 & 0–4 weeks & unvaccinated	41·87 (36·48 to 48·05)	47·08 (41·87 to 52·93)
Wave 3 & 0–4 weeks & 1st vaccine	22·03 (16·08 to 30·17)	19·23 (14·53 to 25·47)
Wave 3 & 0–4 weeks & 2nd vaccine	10·61 (9·62 to 11·7)	10·62 (9·8 to 11·52)
Wave 3 & 0–4 weeks & booster vaccine	3·41 (2·99 to 3·89)	3·5 (3·15 to 3·89)
Wave 3 & 5–26 weeks & unvaccinated	5·38 (4·53 to 6·4)	4·96 (4·21 to 5·85)
Wave 3 & 5–26 weeks & 1st vaccine	4·45 (3·27 to 6·04)	4·76 (3·67 to 6·17)
Wave 3 & 5–26 weeks & 2nd vaccine	2·04 (1·85 to 2·25)	1·88 (1·73 to 2·04)
Wave 3 & 5–26 weeks & booster vaccine	1·56 (1·43 to 1·71)	1·57 (1·46 to 1·69)

Point estimates are derived from a fully adjusted model including covariates: age, ethnicity, deprivation, region, history of asthma, atrial fibrillation, cancer, heart failure, palliative care, cardiovascular disease, diabetes, chronic kidney disease, dementia, coronary heart disease, learning difficulties, stroke and transient ischaemic attack, liver cirrhosis, epilepsy, bipolar disorder, schizophrenia and smoking status.

### Risk of 1st positive SARS-CoV-2 across age groups

**Fig 2:**
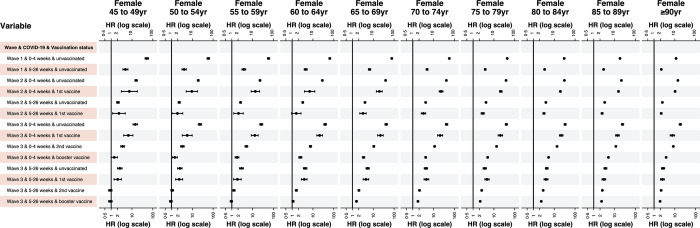
Hazard ratios (and 95% confidence intervals) for wave, COVID-19, and vaccination status terms derived from the Cox Proportional Hazard model denoting the hazard of death amongst females, stratified by age. Hazard ratios for all terms in the multivariable model are provided in the appendices. HR = Hazard ratio. The reference group for a positive COVID-19 test by wave and vaccination status are all those who do not fall into the defined category.

**Fig 3:**
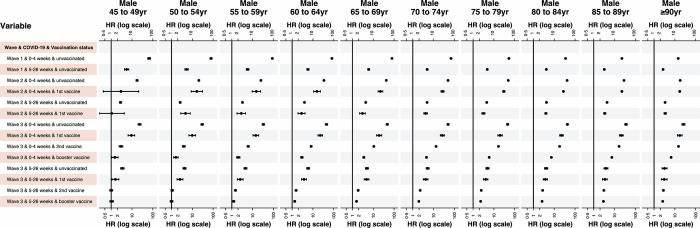
Hazard ratios (and 95% confidence intervals) for wave, COVID-19, and vaccination status terms derived from the Cox Proportional Hazard model denoting the hazard of death amongst males, stratified by age. Hazard ratios for all terms in the multivariable model are provided in the appendices. HR = Hazard ratio. The reference group for a positive COVID-19 test by wave and vaccination status are all those who do not fall into the defined category.

Patient’s age affected the relative risk associated with a positive SARS-CoV-2 test in different ways in successive waves of the pandemic (Figs 2 and 3). In wave one, relative risk generally declined with increasing age. However, in wave two, it generally increased slightly with age, up to age 70–74, and then gradually decreased with increasing age. In wave three, the relative risk generally tended to increase up to age 75–79 and then decreased with increasing age. One exception to this general pattern in wave 3 was in the period 5 to 26 weeks after a positive SARS-CoV-2 test for those with 3 vaccinations. In this situation–although the relative risks in each age group were much lower than those in the period 0 to 4 weeks and for those patients with fewer vaccinations–relative risks generally increased with age to 85–89. Overall, the 95% confidence intervals appeared to become narrower with increasing age.

## Discussion

Our model estimates the relative risk of mortality following a first positive SARS-CoV-2 test by age and sex during the first, second, and third waves of the pandemic in England based on what we understand to be the most complete dataset of individuals in England. The principal finding of this analysis was a reduction in the risk of mortality attributable to COVID-19 over subsequent waves of the pandemic, highlighting the effectiveness of vaccination on mortality in each wave. Our model differentiates the effect of vaccination from the combined effect of waves on other factors, such as SARS-CoV-2 variants, improvements in treatment, and access to care. The model shows the independent effects of SARS-CoV-2 in the presence of a comprehensive list of individual-level characteristics and comorbidities. Our findings provide an immediate and definitive contribution to the literature on COVID-19 risk, derived from an individual-level analysis of the entire English population aged 45 and over during the pandemic. This provides key evidence into the subgroups most vulnerable to mortality following infection, thereby informing future targeted approaches to protecting vulnerable groups from infection, and the clinical management of those who do become infected [3, 19].

There has been a vast amount of research surrounding the COVID-19 pandemic nationally and internationally. Previous studies have included, but are not limited to, examining the impact of COVID-19 in terms of mortality and/or severe outcomes, risk factors associated with outcomes, and effects across time and geographies [2, 4–6, 20–26]. Numerous methodological approaches have been explored in this previous literature, which varied in the study population, how SARS-CoV-2 exposure was measured, and how it was identified. The present study benefits from linking the national SARS-CoV-2 testing data with both death and health records for the English population. In the present study we saw the risk of death from COVID-19 decrease across successive waves in the pandemic. This is consistent with evidence showing reduction in COVID-19 death rates over time in the general population [27]. Reasons for reductions in risk likely include increased availability of treatment, different virus variants, the introduction and rollout of vaccinations, and undetected or unrecorded prior infections. For example, variants that were generally more prevalent in later stages of the pandemic (such as Omicron) were associated with less risk compared with variants that were more prevalent in earlier stages (Delta) [28, 29]. While we did not model vaccine effectiveness per se, we also observed stark reductions in risk of death following vaccination where a SARS-CoV-2 positive test was present, which decreased incrementally with each additional vaccination. This is in line with previous meta-analytic evidence suggesting that vaccination was generally effective against symptomatic COVID-19 and/or serious outcomes such as hospitalisation or death after [30–32], with some findings suggesting a dose-response relationship with vaccination [33, 34]. However, there is likely some waning of vaccine effects over time [31, 34], which we did not consider in our analysis.

Understanding the variation in mortality rates between specific population groups not only guides clinical management and public health strategy during any pandemic, but is also desirable in the aftermath to understand better how expected numbers of deaths have been affected by those with a pre-existing raised level of risk of mortality dying earlier than would otherwise have been the case had the pandemic not occurred [35]. One key example of the latter is the public health need to understand the impact of disruption to a health service following a pandemic. Near real-time monitoring of the impact of such events enables timely response, e.g. allocation of limited resources to groups with the highest need [7]. However, the mortality displacement following a pandemic compromises the accuracy of contemporary methods for real-time monitoring of mortality within the population. The estimates we present in this paper represent the first step in our development of models to make appropriate adjustments for mortality displacement.

Our study population is large and represents the most complete national population dataset available. We have linked a wide range of relevant variables to this population cohort with a high degree of accuracy. The consistency of the modelled estimates (and baseline hazard) for different age-sex groups, which were modelled independently, demonstrates the robustness of the modelling approach in this national population dataset. Our analysis facilitates future work, such as calculating mortality displacement at an individual level, by allowing the point estimates presented in the present paper to be used to inform methods which account for the excess deaths caused by COVID-19 infection during the pandemic. This can ultimately be used to generate more accurate estimates of numbers of deaths that would otherwise have been expected each week, both during a pandemic and subsequently, and therefore improve the monitoring of trends in death rates.

This study has several limitations. Individuals’ comorbidities were defined as at 1^st^ March 2020 and we do not account for individuals who develop comorbidities after this date. Overall, we are likely to overestimate the population as death registration data are subject to a reporting lag and people who are no longer resident in England still being counted in the population database (NIMS) [36]. We only account for the first positive test for SARS-CoV-2 and account for neither the subsequent reduction in risk following infection nor the effects of subsequent infections. Conversely, we treat those who were SARS-CoV-2 positive but did not have a positive test recorded on the NHS website as being free of SARS-CoV-2 and hence included them in the baseline risk. The impact of this is most notable in periods when there was no comprehensive community testing, in particular at the start wave one and later during wave three, after cessation of mandatory testing. During these periods the majority of testing was confined to those being admitted to hospital and hence in the later stages of illness–shortening the survival time from testing to death. What our model also does not show is the interaction of the effect of SARS-CoV-2 with other risk factors. We were unable to model the impact of testing positive for SARS-CoV-2 on mortality for those aged under 45 years due to the sparse number of deaths at this age; nonetheless, the low numbers of deaths in the under 45 age groups mean that the effect of these deaths on future overall mortality rates will be very small. Finally, we did not model the time-dependent relative risk associated with testing positive for SARS-CoV-2 and mortality beyond 26 weeks following infection. Previous research and our results (Figs 2 and 3) suggest that these associations may extend beyond 26 weeks [37], implying longer-term impacts of SARS-CoV-2 infection however these relationships require further investigation and must not be assumed to be causal.

In conclusion, this study provides robust estimates of increased mortality risk among those who did test positive over the first three waves of the COVID-19 pandemic in England. The estimates not only add to the growing understanding of the magnitude of the impact of factors affecting the risk of mortality from COVID-19, but they also provide the first step towards estimating mortality displacement, a measure that is essential to understanding one of the major impacts of the pandemic on subsequent mortality rates in England.

## Supporting information

S1 Appendix Data preparation. (DOCX)

S2 Appendix Statistical modelling. (DOCX)

S3 Appendix Effects of pre-existing health conditions & socio-demographic characteristics on mortality. (DOCX)

S4 Appendix Supplementary data dictionary denoting SNOWMED-CT codes for health condition definitions. (XLSX)

S1 Fig Estimated baseline hazard by sex and five -year age group, ages 45–89*. The baseline hazard estimates the absolute risk of death at any time-point for an individual in whom all Cox model covariates are set to their reference value. Fluctuations in the baseline hazard therefore allow for the identification of periods of excess risk not captured by model covariates. * The open-ended age group 90+ is excluded from the figure as all those who enter this age group ultimately die within the same age-group. Male and female estimates are illustrated discretely as shaded areas (female = orange, male = blue). For each age group, the sex with the higher baseline hazard is evident, as orange or blue filling at the top of the graph, and the corresponding sex with the lower baseline hazard appears grey in the lower part of the graph due to overlapping colours. The fact that the magnitude of the baseline hazards reduced with increasing age likely represents the impact of the defined risk factors, such as pre-existing health conditions, on mortality. The selected risk factors, which are more prevalent amongst older age groups, account for higher levels of mortality at older ages. Fewer risk factors that predominantly relate to death amongst younger age groups were modelled, due to the focus on older age groups where the majority of deaths occur.
(PDF)

S2 Fig Hazard ratios and 95% confidence intervals derived from the Cox Proportional Hazard model denoting the hazard of deaths stratified by age group amongst females. Exact hazard ratios and 95% confidence intervals are presented in S2 Table.
(PDF)

S3 Fig Hazard ratios and 95% confidence intervals derived from the Cox Proportional Hazard model denoting the hazard of deaths stratified by age group amongst males. Exact hazard ratios and 95% confidence intervals are presented in S2 Table.
(PDF)

S1 Table Sociodemographic, health, vaccination, and survival characteristics by gender and positive SARS-CoV-2 test status by 5-year age bands (45+ year olds). Data are presented as n (%) unless otherwise specified. 1st positive COVID-19 tests from 1 March 2020 to 31 March 2022. *Deaths are defined according to censoring.
(DOCX)

S2 Table Hazard ratios and 95% confidence intervals derived from the Cox Proportional Hazard model denoting the hazard of deaths stratified by age group and sex. (DOCX)
