# Supplementary material for: Risk of death in England following a positive SARS-CoV-2 test: A retrospective national cohort study (March 2020 to September 2022)
Source: PLoS One. 2024 Oct 9;19(10):e0304110. doi: 10.1371/journal.pone.0304110 (PMC11463829; doi:10.1371/journal.pone.0304110)
Supplement: S1 Appendix — (DOCX) [file pone.0304110.s001.docx]

# Appendix S1 - Data Preparation

Use of the identifiable datasets for this project was approved by (1) Caldicott Guardians for the UK Health Security Agency (UKHSA) and the Department of Health and Social Care (DHSC), (2) the DHSC Data Protection Officer and Senior Information Risk Owner, when those organisations took over responsibilities from Public Health England (PHE). The weekly excess mortality surveillance process makes use of identifiable data in order to link datasets. Identifiers were removed prior to analysis for this study. Approval to link each of the datasets to each other, for this purpose, was obtained prior to using them from all of the data owners concerned. All approvals are documented and available on request.

## COVID-19 testing data

Surveillance data from England’s SARS-CoV-2 antigen testing programme included national polymerase chain reaction (PCR) and lateral flow antigen testing data for all inpatient (hospital) and community testing since inception in early 2020. Data comprised ‘Pillar 1’ (including all virus testing in PHE laboratories and NHS hospitals) and most ‘Pillar 2’ tests (other virus testing including lighthouse laboratories; public, private and academic laboratories and lateral flow device testing).[^1^](https://www.zotero.org/google-docs/?dfgkhb) Test results are recorded and forwarded from local laboratories to a national dataset, the Second Generation Surveillance System (SGSS), maintained by PHE (until October 2021) and UKHSA. This dataset includes personal identifiable data to allow linkage.

## Dataset preparation and linkage

Each dataset was linked to the National Immunisation Management System (NIMS) denominator through the NHS digital method used to join Hospital Episode Statistics (HES) and Office for National Statistics (ONS) mortality data.[^2–4^](https://www.zotero.org/google-docs/?j1jBMN) This method has previously been used within PHE for enriching datasets with ethnicity during the pandemic. This method was adapted to allow linkage to other datasets and to link a number of fields.

Each dataset was pre-processed by removing additional white space, checking valid entries. Where quality was deemed poor entries were set to NULL. Any duplicates were not removed, but handled after the linkage.

Following preparation, each dataset was passed through the NHS digital match rank process. Using the 8 matched rank criteria set out in the 2015 guide to Linked Mortality Data from Hospital Episode Statistics and the Office for National Statistics.

The match ranks used were:

- Match rank 1: Exact match of DOB, SEX, NHSNO and POSTCODE; if no match is found then
- Match rank 2: Exact match of DOB, SEX, NHSNO; if no match is found then
- Match rank 3: Partial match of DOB and exact match of SEX, NHSNO and
- POSTCODE; if no match found then
- Match rank 4: Partial match of DOB, and exact match of SEX, NHSNO; if no match found then
- Match rank 5: Exact match of POSTCODE and NHSNO; if no match found then
- Match rank 6: Exact match of DOB, SEX and POSTCODE where NHSNO does not contradict the match and DOB is not 1 January and the POSTCODE is not in the 'ignore' list (communal establishments such as hospitals, prisons, army barracks, etc).
- Match rank 7: Exact match of DOB, SEX and POSTCODE where NHSNO does not contradict the match and DOB is not 1 January.
- Match rank 8: Exact match of DOB, SEX and POSTCODE where DOB is not 1 January.

The data to be linked were passed 8 times to the base dataset and the additional columns relating to the lowest rank number selected. Duplicates were handled for each dataset by partitioning the unique id and a rank order created using the newly calculated ascending highest priority. Ascending order dates with ranks equalling 1 were retained thus removing all duplicates and retaining the highest quality matches.

The highest priority column was created based on the highest match rank and order date, unless there was a valid reason to use other fields for example dataset type, valid code as used with ethnicity linkage. The resulting table has a one-to-one match with the additional columns added.

Once all linkage had taken place, a final table removing all personally identifiable fields was created and provided to the analytical team for analysis.

All linkage was completed in a secure SQL database with appropriate access controls. All scripts for dataset preparation and analysis were saved on the PHE internal GitLab repository.

## **Defining comorbidities**

### Hospital Episode Statistics (HES) variables:

Comorbidities that were derived from HES were defined using the following ICD-10 codes:[^5^](https://www.zotero.org/google-docs/?WE6JHR)

- Cardiovascular disease (ICD-10 I00 to I99)
- Diabetes (ICD-10 E10 to E14)
- Chronic kidney disease (ICD-10 N18)
- Chronic lower respiratory disease (ICD-10 J40 to J47)
- Dementia/Alzheimer’s disease (ICD-10 F00 to F03, G30, G31.0, G31.8)

### General Practice Extraction Service (GPES) variables:

The majority of variables that were derived from GPES were based on Quality and Outcomes Framework (QOF) definitions or from previous research which defined and identified conditions that were most at risk from COVID-19 with the exception of diabetes, smoking, or care home resident (see supplementary data dictionary for SNOMED codes).[^6,7^](https://www.zotero.org/google-docs/?tv29N7) The coding for diabetes followed the national audit and smoking status followed the NHS Digital coding.[^8,9^](https://www.zotero.org/google-docs/?hlhZa6) Care home residents were identified by matching postcodes to an establishment lookup (<https://files.digital.nhs.uk/assets/ods/current/ecarehomesite.zip>). Additionally, addresses were checked to determine whether they contained terms like *care, home, nursing, residential, community, elderly, hospice, retirement, general, hospital, centre* to identify persons living in a care home.

### Combined variables:

Where comorbidities were recorded as part of HES and GPES, these were combined. This included diabetes and CKD, where the variables were combined into three distinct levels: no diagnoses, a diagnosis in GPES only, and a diagnosis in HES as a graded severity was assumed with higher risk if a condition was recorded for a hospital admission. Dementia was coded into a binary variable with no diagnosis vs a diagnosis present in either GPES or HES as initial model outcomes showed there were minimal differences in hazard ratios between diagnoses in GPES or HES.

# References

[1 UK Government. About the Data - Coronavirus (COVID-19) in the UK. https://coronavirus.data.gov.uk (accessed Sept 6, 2022).](https://www.zotero.org/google-docs/?MhU6Xu)

[2 NHS Digital. Hospital Episode Statistics (HES). NHS Digital. https://digital.nhs.uk/data-and-information/data-tools-and-services/data-services/hospital-episode-statistics (accessed Nov 8, 2022).](https://www.zotero.org/google-docs/?MhU6Xu)

[3 NHS England. National Vaccination Programmes. https://www.england.nhs.uk/contact-us/privacy-notice/national-flu-vaccination-programme/#immunisation (accessed March 27, 2023).](https://www.zotero.org/google-docs/?MhU6Xu)

[4 NHS Digital. Mortality data review. NHS Digital. https://digital.nhs.uk/coronavirus/coronavirus-data-services-updates/mortality-data-review (accessed March 27, 2023).](https://www.zotero.org/google-docs/?MhU6Xu)

[5 World Health Organization W. International guidelines for certification and classification (coding) of COVID-19 as cause of death. Based on ICD International Statistical Classification of Diseases. 2020.](https://www.zotero.org/google-docs/?MhU6Xu)

[6 Clift AK, Coupland CAC, Keogh RH, *et al.* Living risk prediction algorithm (QCOVID) for risk of hospital admission and mortality from coronavirus 19 in adults: national derivation and validation cohort study. *BMJ* 2020; **371**: m3731.](https://www.zotero.org/google-docs/?MhU6Xu)

[7 NHS Digital. Quality and Outcomes Framework 2021-22. https://qof.digital.nhs.uk/ (accessed March 27, 2023).](https://www.zotero.org/google-docs/?MhU6Xu)

[8 Statistics on Smoking. NHS Digital. https://digital.nhs.uk/data-and-information/publications/statistical/statistics-on-smoking (accessed March 27, 2023).](https://www.zotero.org/google-docs/?MhU6Xu)

[9 NHS Digital. Core National Diabetes Audit. NHS Digital. https://digital.nhs.uk/data-and-information/clinical-audits-and-registries/national-diabetes-audit/core (accessed March 27, 2023).](https://www.zotero.org/google-docs/?MhU6Xu)
