# Supplementary material for: Risk of death in England following a positive SARS-CoV-2 test: A retrospective national cohort study (March 2020 to September 2022)
Source: PLoS One. 2024 Oct 9;19(10):e0304110. doi: 10.1371/journal.pone.0304110 (PMC11463829; doi:10.1371/journal.pone.0304110)
Supplement: S2 Appendix — (DOCX) [file pone.0304110.s002.docx]

# Appendix S2 - Statistical modelling

Separate multivariable Cox Proportional Hazard models were fitted for each 5-year age groups to estimate the relative increase or decrease in the risk of death associated with all exposure variables. Exposures were separated into time-fixed and time-dependent terms. A positive SARS-CoV-2 test was modelled as a time-dependent terms using dummy variables to allow the effects of COVID-19 to vary by time after infection, wave in the pandemic, and vaccination status. Specifically, the hazard associated with a positive SARS-CoV-2 test was modelled as the increase in death rates in the first 4 weeks after a positive test and 5 weeks to 26 weeks, by three time intervals to crudely denote waves in the pandemic: March 2020 to September 2020, October 2020 to March 2021, and April 2021 onwards as well as by vaccination status at the time of a positive test, ranging from 0 to 3 vaccines. A vaccine was defined as taking effect if it was administered 2 weeks prior to a positive test. Due to the timing of vaccine availability and rollout (from December 2020), vaccine status was modelled in such a way that the effect of vaccine was not applied during wave one and vaccines in wave 2 were restricted to 1 vaccine. We performed a sensitivity analysis excluding vaccine status and found comparable results. All other covariates were modelled as time-fixed effects.

In addition to incorporating major health conditions into the models, further comorbidities were selected on the basis of previous research suggesting an increased risk of mortality from COVID-19 and conditions that have a relatively high prevalence and a more immediate impact on mortality in order to capture the risk on death rates within a relatively short follow-up period.[^6^](https://www.zotero.org/google-docs/?okFyca) From the health conditions that were selected a priori, only obesity and hypertension were excluded at the modelling stage. The presence of hypertension and obesity appeared to decrease the hazard ratio, and therefore appeared protective. Obesity was excluded due its association with other health conditions, such as commonly being a precursor to diabetes which was included in the model, are therefore likely led to collinearity. Outcomes in hypertension are highly dependent on the recognition and management - it is likely that well-managed hypertension has a decreased mortality risk compared with undiagnosed hypertension (those who were in the reference group). Missing data on any covariate included in the model was handled by including a “unknown/no record” category.
