# Supplementary material for: Risk of death in England following a positive SARS-CoV-2 test: A retrospective national cohort study (March 2020 to September 2022)
Source: PLoS One. 2024 Oct 9;19(10):e0304110. doi: 10.1371/journal.pone.0304110 (PMC11463829; doi:10.1371/journal.pone.0304110)
Supplement: S3 Appendix — (DOCX) [file pone.0304110.s003.docx]

# Appendix S3 - Effects of pre-existing health conditions & socio-demographic characteristics on mortality

The Cox proportional hazard models quantifying the relative risk of a positive COVID-19 test were adjusted for sociodemographic, clinical, and health characteristics - the estimates for each characteristic represent the relative risk on mortality during the pandemic.

Within each 5-year age group, the relative risk of dying increased with each additional year of age and quintile of deprivation. Having an unknown address, and hence an unknown area deprivation score and region, was associated with a large relative increase in death rates. Relative risks among each known non-white ethnic minority group were lower compared with the white group; however, an unknown ethnicity increased the relative risk to 1.49 (95% CI = 1.41 to 1.57) amongst 60–64-year-old females. Living in London was associated with the lowest relative risk of mortality and living in Yorkshire and the Humber the highest, compared with other regions in England. Across age groups, these patterns, by age, gender and geography were similar. The increased relative risk associated with living in more deprived areas, generally decreased with increasing age, but this trend was more apparent for those areas with higher levels of deprivation.

Having a record of a pre-existing health condition (Table S2) was generally associated with an increased risk of death following a positive COVID-19 test relative to not having such a record. These patterns were remarkably consistent across models by age groups, though relative risks were generally higher at younger age groups and declined with increasing age. However, being classed as clinically vulnerable was associated with an increased relative risk of death in younger age groups. The patterns of increased death rates associated with pre-existing health conditions were similar between the sexes.
