# Supplementary figures and images for: Risk of death in England following a positive SARS-CoV-2 test: A retrospective national cohort study (March 2020 to September 2022)
Source: PLoS One. 2024 Oct 9;19(10):e0304110. doi: 10.1371/journal.pone.0304110 (PMC11463829; doi:10.1371/journal.pone.0304110)

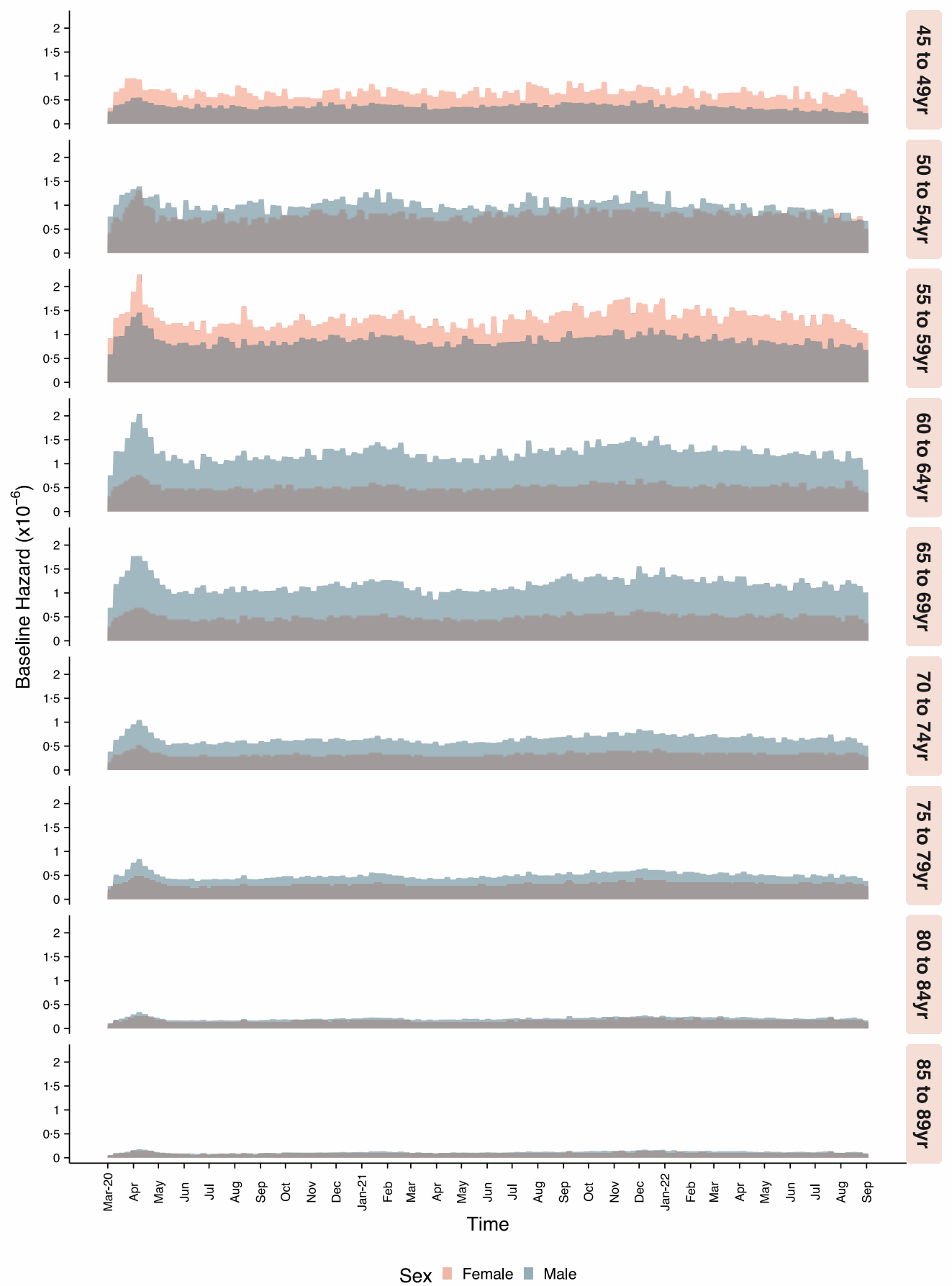

Supplement: S1 Fig — The baseline hazard estimates the absolute risk of death at any time-point for an individual in whom all Cox model covariates are set to their reference value. Fluctuations in the baseline hazard therefore allow for the identification of periods of excess risk not captured by model covariates. * The open-ended age group 90+ is excluded from the figure as all those who enter this age group ultimately die within the same age-group. Male and female estimates are illustrated discretely as shaded areas (female = orange, male = blue). For each age group, the sex with the higher baseline hazard is evident, as orange or blue filling at the top of the graph, and the corresponding sex with the lower baseline hazard appears grey in the lower part of the graph due to overlapping colours. The fact that the magnitude of the baseline hazards reduced with increasing age likely represents the impact of the defined risk factors, such as pre-existing health conditions, on mortality. The selected risk factors, which are more prevalent amongst older age groups, account for higher levels of mortality at older ages. Fewer risk factors that predominantly relate to death amongst younger age groups were modelled, due to the focus on older age groups where the majority of deaths occur. (PDF) [file pone.0304110.s005.pdf]

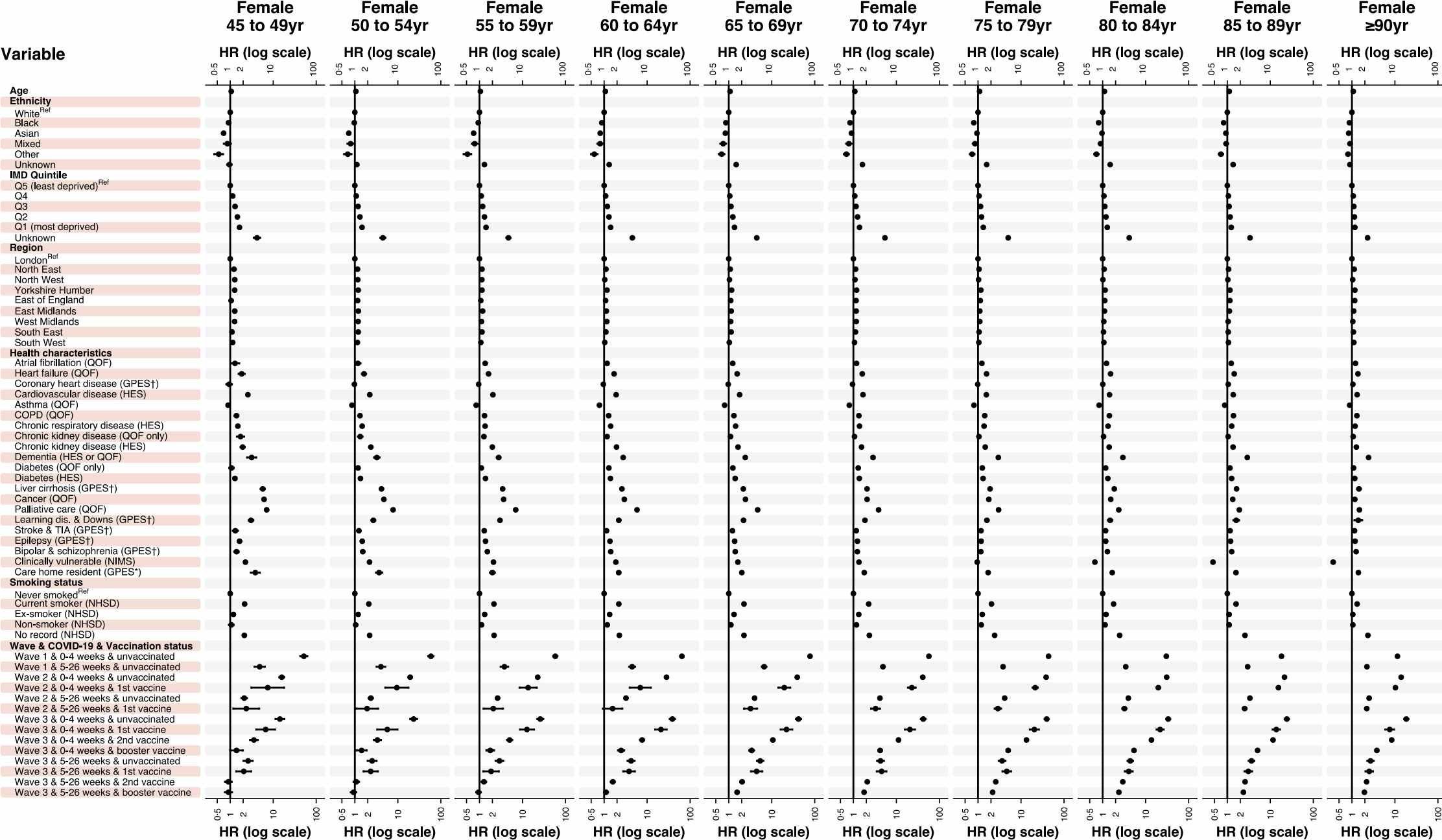

Supplement: S2 Fig — Exact hazard ratios and 95% confidence intervals are presented in S2 Table. (PDF) [file pone.0304110.s006.pdf]

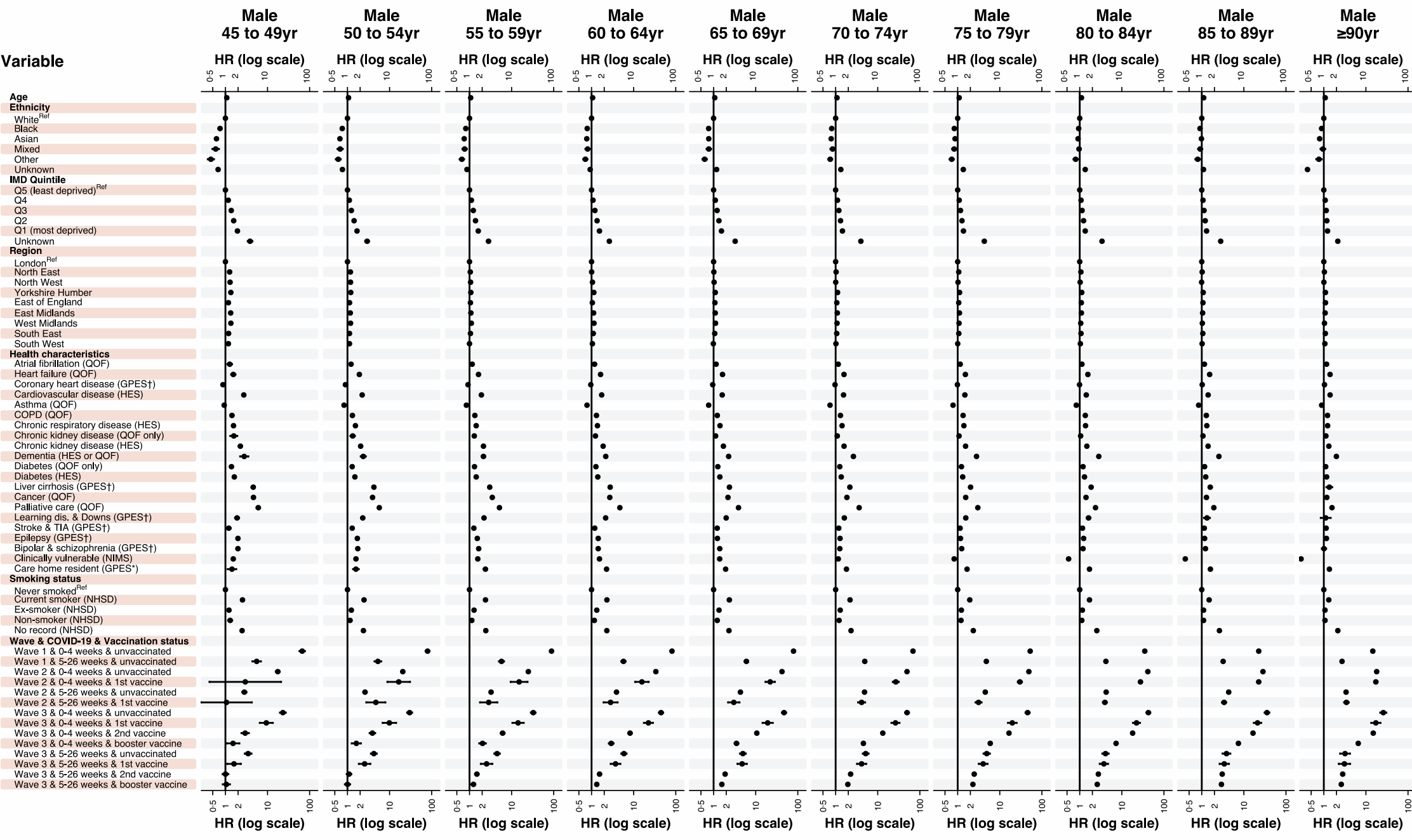

Supplement: S3 Fig — Exact hazard ratios and 95% confidence intervals are presented in S2 Table. (PDF) [file pone.0304110.s007.pdf]
