# Supplementary material for: Risk of death in England following a positive SARS-CoV-2 test: A retrospective national cohort study (March 2020 to September 2022)
Source: PLoS One. 2024 Oct 9;19(10):e0304110. doi: 10.1371/journal.pone.0304110 (PMC11463829; doi:10.1371/journal.pone.0304110)
Supplement: S1 Table — Data are presented as n (%) unless otherwise specified. 1st positive COVID-19 tests from 1 March 2020 to 31 March 2022. *Deaths are defined according to censoring. (DOCX) [file pone.0304110.s008.docx]

**Table S1. Sociodemographic, health, vaccination, and survival characteristics by gender and positive SARS‑CoV‑2 test status by 5-year age bans (45+ year olds)**

|  | **45-49-year-olds** | | | | **50-54-year-olds** | | | |
| --- | --- | --- | --- | --- | --- | --- | --- | --- |
|  | **Female** | | **Male** | | **Female** | | **Male** | |
|  | No positive SARS‑CoV‑2 test | Positive SARS‑CoV‑2 test | No positive SARS‑CoV‑2 test | Positive SARS‑CoV‑2 test | No positive SARS‑CoV‑2 test | Positive SARS‑CoV‑2 test | No positive SARS‑CoV‑2 test | Positive SARS‑CoV‑2 test |
| **n** | 1,349,003 | 633,443.0 | 1,605,100 | 522,394 | 1,496,983 | 568,180 | 1,691,924 | 479,914 |
| **Age (mean(SD)** | 47 (1.41) | 47 (1.41) | 47 (1.42) | 47 (1.41) | 52 (1.41) | 52 (1.41) | 52 (1.41) | 52 (1.41) |
| **Ethnicity** |  |  |  |  |  |  |  |  |
| Asian | 133,452 (9.9) | 51,956 (8.2) | 156,140 (9.7) | 48,034 (9.2) | 104,622 (7.0) | 35,027 (6.2) | 117,264 (6.9) | 33,516 (7.0) |
| Black | 69,560 (5.2) | 23,283 (3.7) | 73,334 (4.6) | 16,952 (3.2) | 72,109 (4.8) | 21,688 (3.8) | 76,719 (4.5) | 16,275 (3.4) |
| Mixed | 19,910 (1.5) | 7,922 (1.3) | 21,595 (1.3) | 6,191 (1.2) | 17,821 (1.2) | 5,848 (1.0) | 18,518 (1.1) | 4,734 (1.0) |
| Other | 26,713 (2.0) | 8,672 (1.4) | 38,273 (2.4) | 9,683 (1.9) | 22,379 (1.5) | 6,092 (1.1) | 29,413 (1.7) | 6,679 (1.4) |
| White | 1,027,994 (76.2) | 526,599 (83.1) | 1,114,170 (69.4) | 408,800 (78.3) | 1,197,398 (80.0) | 482,275 (84.9) | 1,257,424 (74.3) | 391,023 (81.5) |
| Unknown | 71,374 (5.3) | 15,011 (2.4) | 201,588 (12.6) | 32,734 (6.3) | 82,654 (5.5) | 17,250 (3.0) | 192,586 (11.4) | 27,687 (5.8) |
| **IMD Quintile** |  |  |  |  |  |  |  |  |
| 5 (least deprived) | 271,030 (20.1) | 139,405 (22.0) | 297,157 (18.5) | 118,489 (22.7) | 307,041 (20.5) | 119,942 (21.1) | 323,940 (19.1) | 106,539 (22.2) |
| 4 | 264,966 (19.6) | 131,753 (20.8) | 300,009 (18.7) | 109,840 (21.0) | 305,100 (20.4) | 119,085 (21.0) | 329,676 (19.5) | 102,350 (21.3) |
| 3 | 265,897 (19.7) | 125,602 (19.8) | 313,443 (19.5) | 103,132 (19.7) | 302,359 (20.2) | 113,987 (20.1) | 335,336 (19.8) | 96,358 (20.1) |
| 2 | 266,156 (19.7) | 121,203 (19.1) | 332,660 (20.7) | 98,885 (18.9) | 291,509 (19.5) | 110,306 (19.4) | 338,731 (20.0) | 90,613 (18.9) |
| 1 | 262,697 (19.5) | 114,306 (18.0) | 335,041 (20.9) | 90,694 (17.4) | 275,256 (18.4) | 103,982 (18.3) | 336,036 (19.9) | 82,911 (17.3) |
| Unknown | 18,257 (1.4) | 1,174 (0.2) | 26,790 (1.7) | 1,354 (0.3) | 15,718 (1.0) | 878 (0.2) | 28,205 (1.7) | 1,143 (0.2) |
| **Region** |  |  |  |  |  |  |  |  |
| East Midlands | 112,173 (8.3) | 55,728 (8.8) | 129,575 (8.1) | 45,451 (8.7) | 128,939 (8.6) | 51,313 (9.0) | 141,920 (8.4) | 43,134 (9.0) |
| East of England | 157,454 (11.7) | 75,141 (11.9) | 181,777 (11.3) | 61,719 (11.8) | 176,339 (11.8) | 64,978 (11.4) | 194,586 (11.5) | 55,043 (11.5) |
| London | 236,081 (17.5) | 90,528 (14.3) | 301,134 (18.8) | 79,533 (15.2) | 231,512 (15.5) | 75,997 (13.4) | 275,911 (16.3) | 66,521 (13.9) |
| North East | 55,477 (4.1) | 32,162 (5.1) | 65,158 (4.1) | 25,970 (5.0) | 65,897 (4.4) | 31,003 (5.5) | 73,080 (4.3) | 25,314 (5.3) |
| North West | 163,427 (12.1) | 87,323 (13.8) | 197,150 (12.3) | 71,735 (13.7) | 186,410 (12.5) | 82,046 (14.4) | 213,252 (12.6) | 69,193 (14.4) |
| South East | 217,032 (16.1) | 103,641 (16.4) | 247,645 (15.4) | 85,530 (16.4) | 243,420 (16.3) | 89,286 (15.7) | 265,816 (15.7) | 76,374 (15.9) |
| South West | 131,947 (9.8) | 60,362 (9.5) | 147,290 (9.2) | 47,207 (9.0) | 156,750 (10.5) | 54,496 (9.6) | 166,857 (9.9) | 44,549 (9.3) |
| West Midlands | 135,047 (10.0) | 64,843 (10.2) | 163,826 (10.2) | 53,720 (10.3) | 153,227 (10.2) | 59,586 (10.5) | 175,428 (10.4) | 50,109 (10.4) |
| Yorkshire and Humber | 122,108 (9.1) | 62,541 (9.9) | 144,755 (9.0) | 50,175 (9.6) | 138,771 (9.3) | 58,597 (10.3) | 156,869 (9.3) | 48,534 (10.1) |
| Unknown | 18,257 (1.4) | 1,174 (0.2) | 26,790 (1.7) | 1,354 (0.3) | 15,718 (1.0) | 878 (0.2) | 28,205 (1.7) | 1,143 (0.2) |
| **Health Characteristics** |  |  |  |  |  |  |  |  |
| **Atrial fibrillation (QOF)** | 2,593 (0.2) | 1,232 (0.2) | 7,884 (0.5) | 3,334 (0.6) | 5,301 (0.4) | 2,172 (0.4) | 15,385 (0.9) | 5,517 (1.1) |
| **Asthma (QOF)** | 105,985 (7.9) | 56,574 (8.9) | 84,692 (5.3) | 36,515 (7.0) | 124,767 (8.3) | 53,162 (9.4) | 95,808 (5.7) | 34,418 (7.2) |
| **Cancer (QOF)** | 34,043 (2.5) | 15,607 (2.5) | 16,902 (1.1) | 6,066 (1.2) | 60,156 (4.0) | 21,737 (3.8) | 28,696 (1.7) | 8,871 (1.8) |
| **COPD (QOF)** | 10,988 (0.8) | 3,567 (0.6) | 12,876 (0.8) | 2,764 (0.5) | 24,161 (1.6) | 6,887 (1.2) | 27,239 (1.6) | 5,242 (1.1) |
| **Heat failure (QOF)** | 1,978 (0.1) | 763 (0.1) | 4,586 (0.3) | 1,403 (0.3) | 3,541 (0.2) | 1,270 (0.2) | 8,812 (0.5) | 2,444 (0.5) |
| **Palliative care (QOF)** | 2,293 (0.2) | 728 (0.1) | 1,466 (0.1) | 382 (0.1) | 3,809 (0.3) | 1,002 (0.2) | 2,595 (0.2) | 593 (0.1) |
| **Bipolar & schizophrenia (GPES)** | 15,283 (1.1) | 3,935 (0.6) | 20,788 (1.3) | 3,294 (0.6) | 17,556 (1.2) | 3,594 (0.6) | 21,142 (1.2) | 3,150 (0.7) |
| **Coronary heart disease (GPES)** | 5,805 (0.4) | 2,542 (0.4) | 20,330 (1.3) | 6,855 (1.3) | 13,382 (0.9) | 5,093 (0.9) | 44,109 (2.6) | 13,388 (2.8) |
| **Epilepsy (GPES)** | 19,743 (1.5) | 8,078 (1.3) | 22,785 (1.4) | 6,698 (1.3) | 22,231 (1.5) | 7,809 (1.4) | 24,988 (1.5) | 6,651 (1.4) |
| **Learning disability & Downs (GPES)** | 8,502 (0.6) | 2,506 (0.4) | 12,080 (0.8) | 3,070 (0.6) | 8,631 (0.6) | 2,594 (0.5) | 12,427 (0.7) | 3,416 (0.7) |
| **Liver cirrhosis (GPES)** | 2,372 (0.2) | 764 (0.1) | 3,107 (0.2) | 691 (0.1) | 3,617 (0.2) | 1,056 (0.2) | 5,001 (0.3) | 983 (0.2) |
| **Stroke and TIA (GPES)** | 6,925 (0.5) | 2,791 (0.4) | 9,322 (0.6) | 2,834 (0.5) | 13,023 (0.9) | 4,376 (0.8) | 18,308 (1.1) | 4,815 (1.0) |
| **Chronic respiratory disease (HES)** | 73,747 (5.5) | 38,171 (6.0) | 51,683 (3.2) | 20,242 (3.9) | 93,861 (6.3) | 38,925 (6.9) | 66,791 (3.9) | 21,714 (4.5) |
| **Cardiovascular disease (HES)** | 88,595 (6.6) | 43,779 (6.9) | 104,734 (6.5) | 39,661 (7.6) | 144,028 (9.6) | 58,982 (10.4) | 172,711 (10.2) | 56,754 (11.8) |
| **Chronic kidney disease** |  |  |  |  |  |  |  |  |
| No chronic kidney disease | 1,339,845 (99.3) | 629,114 (99.3) | 1,596,166 (99.4) | 518,554 (99.3) | 1,478,290 (98.8) | 560,790 (98.7) | 1,675,219 (99.0) | 473,890 (98.7) |
| Chronic kidney disease (QOF only) | 4,999 (0.4) | 2,234 (0.4) | 4,524 (0.3) | 1,779 (0.3) | 11,528 (0.8) | 4,249 (0.7) | 9,330 (0.6) | 2,987 (0.6) |
| Chronic kidney disease (HES) | 4,159 (0.3) | 2,095 (0.3) | 4,410 (0.3) | 2,061 (0.4) | 7,165 (0.5) | 3,141 (0.6) | 7,375 (0.4) | 3,037 (0.6) |
| **Dementia (QOF or HES)** | 471 (0.0) | 220 (0.0) | 510 (0.0) | 171 (0.0) | 1,111 (0.1) | 370 (0.1) | 1,107 (0.1) | 373 (0.1) |
| **Diabetes** |  |  |  |  |  |  |  |  |
| No diabetes | 1,292,197 (95.8) | 608,376 (96.0) | 1,521,298 (94.8) | 492,460 (94.3) | 1,410,128 (94.2) | 535,431 (94.2) | 1,561,910 (92.3) | 439,139 (91.5) |
| Diabetes (QOF only) | 30,329 (2.2) | 13,017 (2.1) | 51,847 (3.2) | 17,933 (3.4) | 45,137 (3.0) | 16,368 (2.9) | 77,177 (4.6) | 23,363 (4.9) |
| Diabetes (HES) | 26,477 (2.0) | 12,050 (1.9) | 31,955 (2.0) | 12,001 (2.3) | 41,718 (2.8) | 16,381 (2.9) | 52,837 (3.1) | 17,412 (3.6) |
| **Clinically Vulnerable (NIMS** | 94,265 (7.0) | 41,766 (6.6) | 85,352 (5.3) | 29,276 (5.6) | 115,943 (7.7) | 42,135 (7.4) | 106,682 (6.3) | 31,928 (6.7) |
| **Care home (GPES)** | 341 (0.0) | 226 (0.0) | 524 (0.0) | 291 (0.1) | 528 (0.0) | 309 (0.1) | 728 (0.0) | 472 (0.1) |
| **Smoking status (NHSD)** |  |  |  |  |  |  |  |  |
| Current Smoker | 203,347 (15.1) | 70,114 (11.1) | 342,755 (21.4) | 68,267 (13.1) | 228,350 (15.3) | 63,535 (11.2) | 340,881 (20.1) | 56,430 (11.8) |
| Ex-Smoker | 257,414 (19.1) | 147,365 (23.3) | 326,259 (20.3) | 133,566 (25.6) | 293,692 (19.6) | 132,684 (23.4) | 367,214 (21.7) | 127,405 (26.5) |
| Never Smoked | 692,565 (51.3) | 345,812 (54.6) | 638,489 (39.8) | 249,959 (47.8) | 775,727 (51.8) | 311,606 (54.8) | 701,300 (41.4) | 238,049 (49.6) |
| Non-Smoker | 45,191 (3.3) | 23,842 (3.8) | 47,978 (3.0) | 18,231 (3.5) | 49,332 (3.3) | 21,282 (3.7) | 50,753 (3.0) | 17,069 (3.6) |
| No record | 150,486 (11.2) | 46,310 (7.3) | 249,619 (15.6) | 52,371 (10.0) | 149,882 (10.0) | 39,073 (6.9) | 231,776 (13.7) | 40,961 (8.5) |
| **1st Vaccination** | 1,156,579 (85.7) | 593,399 (93.7) | 1,243,876 (77.5) | 482,222 (92.3) | 1,329,930 (88.8) | 538,115 (94.7) | 1,400,461 (82.8) | 451,737 (94.1) |
| **2nd Vaccination** | 1,132,355 (83.9) | 584,517 (92.3) | 1,210,564 (75.4) | 474,366 (90.8) | 1,307,162 (87.3) | 531,762 (93.6) | 1,371,671 (81.1) | 446,351 (93.0) |
| **Booster Vaccination** | 956,167 (70.9) | 488,417 (77.1) | 988,708 (61.6) | 390,210 (74.7) | 1,152,850 (77.0) | 463,557 (81.6) | 1,186,972 (70.2) | 389,128 (81.1) |
| **Deaths*** | 7,296 (0.5) | 1,612 (0.3) | 11,310 (0.7) | 2,201 (0.4) | 11,565 (0.8) | 2,534 (0.4) | 17,988 (1.1) | 3,586 (0.7) |
| **Survival times (median[IQR])** | 130 [130.0, 130.00] | 130 [130.0, 130.00] | 130 [130.0, 130.00] | 130 [130.0, 130.00] | 130 [130.0, 130.00] | 130 [130.0, 130.00] | 130 [130.0, 130.00] | 130 [130.0, 130.00] |
|  |  |  |  |  |  |  |  |  |
|  | **55-59-year-olds** | | | | **60-64-year-olds** | | | |
|  | **Female** | | **Male** | | **Female** | | **Male** | |
|  | No positive SARS‑CoV‑2 test | Positive SARS‑CoV‑2 test | No positive SARS‑CoV‑2 test | Positive SARS‑CoV‑2 test | No positive SARS‑CoV‑2 test | Positive SARS‑CoV‑2 test | No positive SARS‑CoV‑2 test | Positive SARS‑CoV‑2 test |
| **n** | 1,509,790 | 458,253 | 1,629,064 | 404,788.0 | 1,358,744 | 305,486.0 | 1,388,827 | 287,471 |
| **Age (mean(SD)** | 57 (1.41) | 57 (1.41) | 57 (1.41) | 57 (1.41) | 62 (1.41) | 62 (1.40) | 62 (1.41) | 62 (1.40) |
| **Ethnicity** |  |  |  |  |  |  |  |  |
| Asian | 84,941 (5.6) | 23,957 (5.2) | 88,031 (5.4) | 24,342 (6.0) | 79,391 (5.8) | 18,522 (6.1) | 76,330 (5.5) | 19,797 (6.9) |
| Black | 62,118 (4.1) | 16,824 (3.7) | 65,131 (4.0) | 13,210 (3.3) | 40,554 (3.0) | 8,807 (2.9) | 40,277 (2.9) | 7,418 (2.6) |
| Mixed | 14,839 (1.0) | 4,024 (0.9) | 14,879 (0.9) | 3,424 (0.8) | 10,193 (0.8) | 2,124 (0.7) | 9,637 (0.7) | 1,935 (0.7) |
| Other | 18,054 (1.2) | 4,078 (0.9) | 22,036 (1.4) | 4,420 (1.1) | 14,029 (1.0) | 2,558 (0.8) | 15,382 (1.1) | 2,735 (1.0) |
| White | 1,250,206 (82.8) | 395,651 (86.3) | 1,284,535 (78.9) | 340,735 (84.2) | 1,145,054 (84.3) | 264,500 (86.6) | 1,130,858 (81.4) | 244,431 (85.0) |
| Unknown | 79,632 (5.3) | 13,719 (3.0) | 154,452 (9.5) | 18,657 (4.6) | 69,523 (5.1) | 8,975 (2.9) | 116,343 (8.4) | 11,155 (3.9) |
| **IMD Quintile** |  |  |  |  |  |  |  |  |
| 5 (least deprived) | 316,166 (20.9) | 95,962 (20.9) | 321,879 (19.8) | 89,167 (22.0) | 287,374 (21.1) | 64,418 (21.1) | 282,305 (20.3) | 63,245 (22.0) |
| 4 | 317,358 (21.0) | 96,351 (21.0) | 329,297 (20.2) | 87,303 (21.6) | 291,672 (21.5) | 65,339 (21.4) | 288,334 (20.8) | 62,429 (21.7) |
| 3 | 310,693 (20.6) | 93,497 (20.4) | 329,281 (20.2) | 81,906 (20.2) | 282,852 (20.8) | 62,388 (20.4) | 284,722 (20.5) | 58,570 (20.4) |
| 2 | 286,573 (19.0) | 88,426 (19.3) | 316,930 (19.5) | 76,796 (19.0) | 254,522 (18.7) | 58,465 (19.1) | 263,149 (18.9) | 53,826 (18.7) |
| 1 | 263,623 (17.5) | 83,295 (18.2) | 305,509 (18.8) | 68,792 (17.0) | 228,670 (16.8) | 54,476 (17.8) | 247,276 (17.8) | 48,868 (17.0) |
| Unknown | 15,377 (1.0) | 722 (0.2) | 26,168 (1.6) | 824 (0.2) | 13,654 (1.0) | 400 (0.1) | 23,041 (1.7) | 533 (0.2) |
| **Region** |  |  |  |  |  |  |  |  |
| East Midlands | 132,367 (8.8) | 40,892 (8.9) | 139,881 (8.6) | 36,470 (9.0) | 120,155 (8.8) | 26,503 (8.7) | 120,990 (8.7) | 25,331 (8.8) |
| East of England | 177,089 (11.7) | 51,821 (11.3) | 187,614 (11.5) | 45,827 (11.3) | 159,364 (11.7) | 34,425 (11.3) | 160,643 (11.6) | 32,245 (11.2) |
| London | 210,713 (14.0) | 59,419 (13.0) | 235,227 (14.4) | 54,030 (13.3) | 172,644 (12.7) | 39,202 (12.8) | 178,652 (12.9) | 37,717 (13.1) |
| North East | 72,838 (4.8) | 26,531 (5.8) | 76,380 (4.7) | 22,760 (5.6) | 70,783 (5.2) | 18,332 (6.0) | 70,877 (5.1) | 16,869 (5.9) |
| North West | 194,297 (12.9) | 68,430 (14.9) | 212,544 (13.0) | 60,341 (14.9) | 177,459 (13.1) | 45,791 (15.0) | 181,887 (13.1) | 43,384 (15.1) |
| South East | 245,106 (16.2) | 71,142 (15.5) | 259,762 (15.9) | 63,747 (15.7) | 218,520 (16.1) | 47,364 (15.5) | 221,187 (15.9) | 44,640 (15.5) |
| South West | 165,649 (11.0) | 44,338 (9.7) | 170,733 (10.5) | 38,289 (9.5) | 154,282 (11.4) | 30,150 (9.9) | 153,255 (11.0) | 27,510 (9.6) |
| West Midlands | 153,394 (10.2) | 47,532 (10.4) | 166,645 (10.2) | 41,345 (10.2) | 138,796 (10.2) | 31,638 (10.4) | 143,241 (10.3) | 29,440 (10.2) |
| Yorkshire and Humber | 142,960 (9.5) | 47,426 (10.3) | 154,110 (9.5) | 41,155 (10.2) | 133,087 (9.8) | 31,681 (10.4) | 135,054 (9.7) | 29,802 (10.4) |
| Unknown | 15,377 (1.0) | 722 (0.2) | 26,168 (1.6) | 824 (0.2) | 13,654 (1.0) | 400 (0.1) | 23,041 (1.7) | 533 (0.2) |
| **Health Characteristics** |  |  |  |  |  |  |  |  |
| **Atrial fibrillation (QOF)** | 10,037 (0.7) | 3,367 (0.7) | 26,822 (1.6) | 7,969 (2.0) | 17,140 (1.3) | 4,335 (1.4) | 40,856 (2.9) | 9,897 (3.4) |
| **Asthma (QOF)** | 131,325 (8.7) | 44,971 (9.8) | 98,039 (6.0) | 30,627 (7.6) | 123,251 (9.1) | 32,136 (10.5) | 86,759 (6.2) | 22,680 (7.9) |
| **Cancer (QOF)** | 82,800 (5.5) | 24,222 (5.3) | 46,568 (2.9) | 12,561 (3.1) | 94,155 (6.9) | 21,101 (6.9) | 67,160 (4.8) | 15,425 (5.4) |
| **COPD (QOF)** | 40,769 (2.7) | 9,923 (2.2) | 43,329 (2.7) | 8,231 (2.0) | 56,871 (4.2) | 11,282 (3.7) | 59,299 (4.3) | 10,558 (3.7) |
| **Heat failure (QOF)** | 5,955 (0.4) | 1,735 (0.4) | 14,276 (0.9) | 3,690 (0.9) | 8,604 (0.6) | 2,122 (0.7) | 20,177 (1.5) | 4,496 (1.6) |
| **Palliative care (QOF)** | 5,340 (0.4) | 1,274 (0.3) | 4,122 (0.3) | 931 (0.2) | 6,690 (0.5) | 1,375 (0.5) | 6,048 (0.4) | 1,213 (0.4) |
| **Bipolar & schizophrenia (GPES)** | 17,546 (1.2) | 3,490 (0.8) | 19,321 (1.2) | 3,019 (0.7) | 14,598 (1.1) | 2,520 (0.8) | 14,159 (1.0) | 2,425 (0.8) |
| **Coronary heart disease (GPES)** | 24,853 (1.6) | 7,735 (1.7) | 76,573 (4.7) | 21,123 (5.2) | 37,859 (2.8) | 9,373 (3.1) | 106,901 (7.7) | 25,129 (8.7) |
| **Epilepsy (GPES)** | 22,890 (1.5) | 6,636 (1.4) | 24,783 (1.5) | 6,044 (1.5) | 19,962 (1.5) | 4,498 (1.5) | 20,864 (1.5) | 4,622 (1.6) |
| **Learning disability & Downs (GPES)** | 7,992 (0.5) | 2,447 (0.5) | 11,576 (0.7) | 3,070 (0.8) | 5,553 (0.4) | 1,697 (0.6) | 7,851 (0.6) | 2,209 (0.8) |
| **Liver cirrhosis (GPES)** | 4,921 (0.3) | 1,227 (0.3) | 6,379 (0.4) | 1,337 (0.3) | 5,507 (0.4) | 1,205 (0.4) | 6,586 (0.5) | 1,373 (0.5) |
| **Stroke and TIA (GPES)** | 19,785 (1.3) | 5,700 (1.2) | 29,871 (1.8) | 7,215 (1.8) | 26,375 (1.9) | 5,842 (1.9) | 40,967 (2.9) | 8,828 (3.1) |
| **Chronic respiratory disease (HES)** | 113,666 (7.5) | 37,842 (8.3) | 85,445 (5.2) | 24,765 (6.1) | 113,789 (8.4) | 28,655 (9.4) | 89,050 (6.4) | 21,603 (7.5) |
| **Cardiovascular disease (HES)** | 206,563 (13.7) | 68,371 (14.9) | 257,412 (15.8) | 75,629 (18.7) | 241,512 (17.8) | 60,771 (19.9) | 304,021 (21.9) | 74,431 (25.9) |
| **Chronic kidney disease** |  |  |  |  |  |  |  |  |
| No chronic kidney disease | 1,477,079 (97.8) | 447,745 (97.7) | 1,601,189 (98.3) | 395,786 (97.8) | 1,309,576 (96.4) | 293,223 (96.0) | 1,347,370 (97.0) | 276,589 (96.2) |
| Chronic kidney disease (QOF only) | 21,419 (1.4) | 6,622 (1.4) | 16,606 (1.0) | 4,771 (1.2) | 33,242 (2.4) | 7,806 (2.6) | 24,974 (1.8) | 5,713 (2.0) |
| Chronic kidney disease (HES) | 11,292 (0.7) | 3,886 (0.8) | 11,269 (0.7) | 4,231 (1.0) | 15,926 (1.2) | 4,457 (1.5) | 16,483 (1.2) | 5,169 (1.8) |
| **Dementia (QOF or HES)** | 2,227 (0.1) | 764 (0.2) | 2,319 (0.1) | 793 (0.2) | 3,865 (0.3) | 1,234 (0.4) | 4,436 (0.3) | 1,441 (0.5) |
| **Diabetes** |  |  |  |  |  |  |  |  |
| No diabetes | 1,391,911 (92.2) | 421,581 (92.0) | 1,457,422 (89.5) | 356,124 (88.0) | 1,221,423 (89.9) | 272,119 (89.1) | 1,193,173 (85.9) | 240,164 (83.5) |
| Diabetes (QOF only) | 56,683 (3.8) | 16,448 (3.6) | 90,678 (5.6) | 23,978 (5.9) | 64,802 (4.8) | 14,509 (4.7) | 98,998 (7.1) | 21,948 (7.6) |
| Diabetes (HES) | 61,196 (4.1) | 20,224 (4.4) | 80,964 (5.0) | 24,686 (6.1) | 72,519 (5.3) | 18,858 (6.2) | 96,656 (7.0) | 25,359 (8.8) |
| **Clinically Vulnerable (NIMS** | 129,316 (8.6) | 39,095 (8.5) | 118,032 (7.2) | 32,251 (8.0) | 128,987 (9.5) | 31,864 (10.4) | 120,958 (8.7) | 29,627 (10.3) |
| **Care home (GPES)** | 794 (0.1) | 511 (0.1) | 1,142 (0.1) | 760 (0.2) | 1,096 (0.1) | 811 (0.3) | 1,426 (0.1) | 1,022 (0.4) |
| **Smoking status (NHSD)** |  |  |  |  |  |  |  |  |
| Current Smoker | 213,780 (14.2) | 47,026 (10.3) | 292,200 (17.9) | 41,340 (10.2) | 166,748 (12.3) | 26,138 (8.6) | 213,944 (15.4) | 25,059 (8.7) |
| Ex-Smoker | 302,460 (20.0) | 106,655 (23.3) | 382,217 (23.5) | 112,741 (27.9) | 307,698 (22.6) | 78,679 (25.8) | 379,719 (27.3) | 90,981 (31.6) |
| Never Smoked | 799,661 (53.0) | 256,679 (56.0) | 707,818 (43.4) | 206,675 (51.1) | 713,370 (52.5) | 169,723 (55.6) | 600,681 (43.3) | 142,142 (49.4) |
| Non-Smoker | 48,386 (3.2) | 16,551 (3.6) | 48,358 (3.0) | 13,873 (3.4) | 40,659 (3.0) | 10,193 (3.3) | 39,206 (2.8) | 9,239 (3.2) |
| No record | 145,503 (9.6) | 31,342 (6.8) | 198,471 (12.2) | 30,159 (7.5) | 130,269 (9.6) | 20,753 (6.8) | 155,277 (11.2) | 20,050 (7.0) |
| **1st Vaccination** | 1,363,929 (90.3) | 435,924 (95.1) | 1,400,956 (86.0) | 384,619 (95.0) | 1,241,251 (91.4) | 291,760 (95.5) | 1,227,660 (88.4) | 273,920 (95.3) |
| **2nd Vaccination** | 1,344,052 (89.0) | 431,481 (94.2) | 1,378,158 (84.6) | 380,901 (94.1) | 1,225,111 (90.2) | 289,057 (94.6) | 1,210,882 (87.2) | 271,511 (94.4) |
| **Booster Vaccination** | 1,213,167 (80.4) | 385,554 (84.1) | 1,233,104 (75.7) | 343,257 (84.8) | 1,133,879 (83.5) | 266,100 (87.1) | 1,118,034 (80.5) | 252,442 (87.8) |
| **Deaths*** | 16,876 (1.1) | 3,565 (0.8) | 25,855 (1.6) | 5,753 (1.4) | 22,822 (1.7) | 4,964 (1.6) | 33,466 (2.4) | 8,011 (2.8) |
| **Survival times (median[IQR])** | 130 [130.0, 130.00] | 130 [130.0, 130.00] | 130 [130.0, 130.00] | 130 [130.0, 130.00] | 130 [130.0, 130.00] | 130 [130.0, 130.00] | 130 [130.0, 130.00] | 130 [130.0, 130.00] |
|  |  |  |  |  |  |  |  |  |
|  | **65-69-year-olds** | | | | **70-74-year-olds** | | | |
|  | **Female** | | **Male** | | **Female** | | **Male** | |
|  | No positive SARS‑CoV‑2 test | Positive SARS‑CoV‑2 test | No positive SARS‑CoV‑2 test | Positive SARS‑CoV‑2 test | No positive SARS‑CoV‑2 test | Positive SARS‑CoV‑2 test | No positive SARS‑CoV‑2 test | Positive SARS‑CoV‑2 test |
| **n** | 1,274,145 | 197,251 | 1,213,395 | 199,829 | 1,320,172 | 160,717.0 | 1,198,722 | 169,772.0 |
| **Age (mean(SD)** | 67 (1.42) | 67 (1.41) | 67 (1.42) | 67 (1.41) | 72 (1.37) | 72 (1.37) | 72 (1.36) | 72 (1.36) |
| **Ethnicity** |  |  |  |  |  |  |  |  |
| Asian | 69,059 (5.4) | 12,887 (6.5) | 62,163 (5.1) | 14,062 (7.0) | 49,784 (3.8) | 7,695 (4.8) | 38,659 (3.2) | 7,192 (4.2) |
| Black | 25,481 (2.0) | 3,811 (1.9) | 21,092 (1.7) | 3,159 (1.6) | 18,296 (1.4) | 2,148 (1.3) | 11,860 (1.0) | 1,680 (1.0) |
| Mixed | 7,170 (0.6) | 1,039 (0.5) | 6,129 (0.5) | 1,003 (0.5) | 5,300 (0.4) | 624 (0.4) | 3,993 (0.3) | 583 (0.3) |
| Other | 11,059 (0.9) | 1,394 (0.7) | 10,175 (0.8) | 1,463 (0.7) | 8,291 (0.6) | 873 (0.5) | 7,165 (0.6) | 887 (0.5) |
| White | 1,104,874 (86.7) | 173,478 (87.9) | 1,041,654 (85.8) | 174,806 (87.5) | 1,188,018 (90.0) | 146,413 (91.1) | 1,083,158 (90.4) | 156,150 (92.0) |
| Unknown | 56,502 (4.4) | 4,642 (2.4) | 72,182 (5.9) | 5,336 (2.7) | 50,483 (3.8) | 2,964 (1.8) | 53,887 (4.5) | 3,280 (1.9) |
| **IMD Quintile** |  |  |  |  |  |  |  |  |
| 5 (least deprived) | 282,195 (22.1) | 45,071 (22.8) | 259,472 (21.4) | 46,203 (23.1) | 315,465 (23.9) | 38,000 (23.6) | 279,660 (23.3) | 40,925 (24.1) |
| 4 | 283,112 (22.2) | 43,699 (22.2) | 262,818 (21.7) | 44,782 (22.4) | 305,343 (23.1) | 36,272 (22.6) | 275,662 (23.0) | 39,240 (23.1) |
| 3 | 268,905 (21.1) | 40,501 (20.5) | 253,518 (20.9) | 40,526 (20.3) | 279,003 (21.1) | 32,797 (20.4) | 254,585 (21.2) | 34,433 (20.3) |
| 2 | 231,399 (18.2) | 35,581 (18.0) | 222,215 (18.3) | 35,893 (18.0) | 227,926 (17.3) | 28,311 (17.6) | 206,892 (17.3) | 29,400 (17.3) |
| 1 | 196,871 (15.5) | 32,162 (16.3) | 198,681 (16.4) | 32,114 (16.1) | 182,402 (13.8) | 25,157 (15.7) | 169,267 (14.1) | 25,569 (15.1) |
| Unknown | 11,663 (0.9) | 237 (0.1) | 16,691 (1.4) | 311 (0.2) | 10,033 (0.8) | 180 (0.1) | 12,656 (1.1) | 205 (0.1) |
| **Region** |  |  |  |  |  |  |  |  |
| East Midlands | 115,591 (9.1) | 17,001 (8.6) | 110,679 (9.1) | 18,020 (9.0) | 120,966 (9.2) | 14,270 (8.9) | 111,500 (9.3) | 15,100 (8.9) |
| East of England | 153,763 (12.1) | 22,262 (11.3) | 143,177 (11.8) | 22,418 (11.2) | 164,947 (12.5) | 18,345 (11.4) | 148,548 (12.4) | 19,621 (11.6) |
| London | 143,537 (11.3) | 24,754 (12.5) | 132,725 (10.9) | 24,359 (12.2) | 130,760 (9.9) | 18,657 (11.6) | 112,292 (9.4) | 18,672 (11.0) |
| North East | 66,044 (5.2) | 11,584 (5.9) | 63,715 (5.3) | 11,741 (5.9) | 66,835 (5.1) | 9,420 (5.9) | 61,471 (5.1) | 9,823 (5.8) |
| North West | 166,283 (13.1) | 29,493 (15.0) | 161,315 (13.3) | 30,350 (15.2) | 171,566 (13.0) | 24,623 (15.3) | 158,097 (13.2) | 26,212 (15.4) |
| South East | 206,816 (16.2) | 31,358 (15.9) | 193,479 (15.9) | 31,126 (15.6) | 224,193 (17.0) | 26,136 (16.3) | 199,493 (16.6) | 27,874 (16.4) |
| South West | 151,721 (11.9) | 20,203 (10.2) | 142,270 (11.7) | 19,836 (9.9) | 162,570 (12.3) | 16,427 (10.2) | 149,009 (12.4) | 17,625 (10.4) |
| West Midlands | 133,460 (10.5) | 20,319 (10.3) | 127,935 (10.5) | 21,195 (10.6) | 137,484 (10.4) | 16,432 (10.2) | 126,028 (10.5) | 17,610 (10.4) |
| Yorkshire and Humber | 125,267 (9.8) | 20,040 (10.2) | 121,409 (10.0) | 20,473 (10.2) | 130,818 (9.9) | 16,227 (10.1) | 119,628 (10.0) | 17,030 (10.0) |
| Unknown | 11,663 (0.9) | 237 (0.1) | 16,691 (1.4) | 311 (0.2) | 10,033 (0.8) | 180 (0.1) | 12,656 (1.1) | 205 (0.1) |
| **Health Characteristics** |  |  |  |  |  |  |  |  |
| **Atrial fibrillation (QOF)** | 30,122 (2.4) | 5,567 (2.8) | 61,895 (5.1) | 12,002 (6.0) | 56,238 (4.3) | 8,255 (5.1) | 98,761 (8.2) | 16,804 (9.9) |
| **Asthma (QOF)** | 117,006 (9.2) | 22,298 (11.3) | 78,389 (6.5) | 16,831 (8.4) | 119,741 (9.1) | 18,402 (11.4) | 76,011 (6.3) | 13,741 (8.1) |
| **Cancer (QOF)** | 110,501 (8.7) | 17,464 (8.9) | 96,244 (7.9) | 18,087 (9.1) | 139,811 (10.6) | 18,046 (11.2) | 142,458 (11.9) | 22,479 (13.2) |
| **COPD (QOF)** | 71,555 (5.6) | 11,007 (5.6) | 78,244 (6.4) | 12,665 (6.3) | 88,947 (6.7) | 12,691 (7.9) | 96,911 (8.1) | 15,635 (9.2) |
| **Heat failure (QOF)** | 12,939 (1.0) | 2,435 (1.2) | 27,664 (2.3) | 5,268 (2.6) | 21,620 (1.6) | 3,578 (2.2) | 39,992 (3.3) | 7,185 (4.2) |
| **Palliative care (QOF)** | 8,276 (0.6) | 1,507 (0.8) | 8,165 (0.7) | 1,625 (0.8) | 12,001 (0.9) | 2,348 (1.5) | 11,940 (1.0) | 2,335 (1.4) |
| **Bipolar & schizophrenia (GPES)** | 12,181 (1.0) | 2,133 (1.1) | 10,726 (0.9) | 1,951 (1.0) | 11,043 (0.8) | 2,149 (1.3) | 8,294 (0.7) | 1,771 (1.0) |
| **Coronary heart disease (GPES)** | 52,544 (4.1) | 9,550 (4.8) | 133,306 (11.0) | 25,668 (12.8) | 78,135 (5.9) | 11,757 (7.3) | 174,023 (14.5) | 28,787 (17.0) |
| **Epilepsy (GPES)** | 18,308 (1.4) | 3,190 (1.6) | 17,820 (1.5) | 3,400 (1.7) | 18,120 (1.4) | 2,792 (1.7) | 17,080 (1.4) | 3,024 (1.8) |
| **Learning disability & Downs (GPES)** | 3,743 (0.3) | 1,264 (0.6) | 5,085 (0.4) | 1,527 (0.8) | 2,633 (0.2) | 987 (0.6) | 3,238 (0.3) | 1,244 (0.7) |
| **Liver cirrhosis (GPES)** | 5,806 (0.5) | 1,052 (0.5) | 6,326 (0.5) | 1,172 (0.6) | 6,123 (0.5) | 1,016 (0.6) | 5,530 (0.5) | 1,039 (0.6) |
| **Stroke and TIA (GPES)** | 35,476 (2.8) | 6,215 (3.2) | 52,909 (4.4) | 9,587 (4.8) | 52,807 (4.0) | 8,063 (5.0) | 72,584 (6.1) | 12,299 (7.2) |
| **Chronic respiratory disease (HES)** | 124,610 (9.8) | 23,878 (12.1) | 102,397 (8.4) | 20,972 (10.5) | 150,183 (11.4) | 24,604 (15.3) | 122,553 (10.2) | 23,007 (13.6) |
| **Cardiovascular disease (HES)** | 299,601 (23.5) | 54,072 (27.4) | 359,938 (29.7) | 71,176 (35.6) | 410,853 (31.1) | 60,129 (37.4) | 450,981 (37.6) | 76,553 (45.1) |
| **Chronic kidney disease** |  |  |  |  |  |  |  |  |
| No chronic kidney disease | 1,194,134 (93.7) | 182,678 (92.6) | 1,146,716 (94.5) | 185,884 (93.0) | 1,179,546 (89.3) | 140,484 (87.4) | 1,080,762 (90.2) | 149,880 (88.3) |
| Chronic kidney disease (QOF only) | 55,462 (4.4) | 8,979 (4.6) | 41,223 (3.4) | 7,412 (3.7) | 96,868 (7.3) | 12,039 (7.5) | 73,891 (6.2) | 10,672 (6.3) |
| Chronic kidney disease (HES) | 24,549 (1.9) | 5,594 (2.8) | 25,456 (2.1) | 6,533 (3.3) | 43,758 (3.3) | 8,194 (5.1) | 44,069 (3.7) | 9,220 (5.4) |
| **Dementia (QOF or HES)** | 6,924 (0.5) | 2,240 (1.1) | 7,538 (0.6) | 2,516 (1.3) | 16,690 (1.3) | 5,660 (3.5) | 15,663 (1.3) | 5,348 (3.2) |
| **Diabetes** |  |  |  |  |  |  |  |  |
| No diabetes | 1,123,002 (88.1) | 169,774 (86.1) | 1,004,165 (82.8) | 159,536 (79.8) | 1,145,552 (86.8) | 134,742 (83.8) | 968,899 (80.8) | 131,997 (77.7) |
| Diabetes (QOF only) | 67,827 (5.3) | 10,370 (5.3) | 96,623 (8.0) | 15,971 (8.0) | 72,222 (5.5) | 8,270 (5.1) | 94,669 (7.9) | 12,316 (7.3) |
| Diabetes (HES) | 83,316 (6.5) | 17,107 (8.7) | 112,607 (9.3) | 24,322 (12.2) | 102,398 (7.8) | 17,705 (11.0) | 135,154 (11.3) | 25,459 (15.0) |
| **Clinically Vulnerable (NIMS** | 131,045 (10.3) | 26,031 (13.2) | 128,733 (10.6) | 27,631 (13.8) | 154,303 (11.7) | 27,951 (17.4) | 169,976 (14.2) | 33,586 (19.8) |
| **Care home (GPES)** | 1,728 (0.1) | 1,326 (0.7) | 2,071 (0.2) | 1,550 (0.8) | 3,788 (0.3) | 3,118 (1.9) | 3,618 (0.3) | 2,941 (1.7) |
| **Smoking status (NHSD)** |  |  |  |  |  |  |  |  |
| Current Smoker | 127,211 (10.0) | 12,317 (6.2) | 152,827 (12.6) | 14,295 (7.2) | 102,698 (7.8) | 8,438 (5.3) | 111,155 (9.3) | 9,952 (5.9) |
| Ex-Smoker | 330,605 (25.9) | 56,537 (28.7) | 412,781 (34.0) | 75,441 (37.8) | 368,986 (27.9) | 50,105 (31.2) | 473,975 (39.5) | 72,751 (42.9) |
| Never Smoked | 661,171 (51.9) | 108,250 (54.9) | 493,671 (40.7) | 90,514 (45.3) | 692,991 (52.5) | 85,348 (53.1) | 470,394 (39.2) | 69,778 (41.1) |
| Non-Smoker | 36,196 (2.8) | 6,176 (3.1) | 31,833 (2.6) | 5,737 (2.9) | 35,989 (2.7) | 4,706 (2.9) | 29,912 (2.5) | 4,718 (2.8) |
| No record | 118,962 (9.3) | 13,971 (7.1) | 122,283 (10.1) | 13,842 (6.9) | 119,508 (9.1) | 12,120 (7.5) | 113,286 (9.5) | 12,573 (7.4) |
| **1st Vaccination** | 1,178,744 (92.5) | 188,169 (95.4) | 1,100,381 (90.7) | 189,661 (94.9) | 1,237,953 (93.8) | 151,986 (94.6) | 1,107,237 (92.4) | 158,813 (93.5) |
| **2nd Vaccination** | 1,165,584 (91.5) | 186,411 (94.5) | 1,087,335 (89.6) | 187,986 (94.1) | 1,225,742 (92.8) | 150,281 (93.5) | 1,095,158 (91.4) | 156,934 (92.4) |
| **Booster Vaccination** | 1,104,652 (86.7) | 175,857 (89.2) | 1,030,480 (84.9) | 178,556 (89.4) | 1,173,911 (88.9) | 142,451 (88.6) | 1,048,848 (87.5) | 149,594 (88.1) |
| **Deaths*** | 31,781 (2.5) | 6,809 (3.5) | 45,926 (3.8) | 10,508 (5.3) | 52,046 (3.9) | 10,961 (6.8) | 69,248 (5.8) | 16,480 (9.7) |
| **Survival times (median[IQR])** | 130 [130.0, 130.00] | 130 [130.0, 130.00] | 130 [130.0, 130.00] | 130 [130.0, 130.00] | 130 [130.0, 130.00] | 130 [130.0, 130.00] | 130 [130.0, 130.00] | 130 [130.0, 130.00] |
|  |  |  |  |  |  |  |  |  |
|  | **75-79-year-olds** | | | | **80-84-year-olds** | | | |
|  | **Female** | | **Male** | | **Female** | | **Male** | |
|  | No positive SARS‑CoV‑2 test | Positive SARS‑CoV‑2 test | No positive SARS‑CoV‑2 test | Positive SARS‑CoV‑2 test | No positive SARS‑CoV‑2 test | Positive SARS‑CoV‑2 test | No positive SARS‑CoV‑2 test | Positive SARS‑CoV‑2 test |
| **n** | 975,407 | 107,910 | 837,074 | 111,090 | 731,798 | 89,252.0 | 574,480 | 78,278.0 |
| **Age (mean(SD)** | 77 (1.41) | 77 (1.41) | 77 (1.40) | 77 (1.40) | 82 (1.40) | 82 (1.41) | 82 (1.40) | 82 (1.40) |
| **Ethnicity** |  |  |  |  |  |  |  |  |
| Asian | 32,197 (3.3) | 4,900 (4.5) | 28,076 (3.4) | 5,083 (4.6) | 23,770 (3.2) | 3,629 (4.1) | 22,070 (3.8) | 4,069 (5.2) |
| Black | 17,291 (1.8) | 2,079 (1.9) | 11,035 (1.3) | 1,609 (1.4) | 14,072 (1.9) | 1,839 (2.1) | 10,621 (1.8) | 1,788 (2.3) |
| Mixed | 3,768 (0.4) | 391 (0.4) | 2,798 (0.3) | 386 (0.3) | 2,574 (0.4) | 325 (0.4) | 1,931 (0.3) | 305 (0.4) |
| Other | 4,905 (0.5) | 410 (0.4) | 4,055 (0.5) | 427 (0.4) | 3,148 (0.4) | 275 (0.3) | 2,182 (0.4) | 248 (0.3) |
| White | 885,196 (90.8) | 98,655 (91.4) | 760,777 (90.9) | 102,031 (91.8) | 666,835 (91.1) | 82,240 (92.1) | 519,083 (90.4) | 71,106 (90.8) |
| Unknown | 32,050 (3.3) | 1,475 (1.4) | 30,333 (3.6) | 1,554 (1.4) | 21,399 (2.9) | 944 (1.1) | 18,593 (3.2) | 762 (1.0) |
| **IMD Quintile** |  |  |  |  |  |  |  |  |
| 5 (least deprived) | 235,159 (24.1) | 23,738 (22.0) | 201,440 (24.1) | 25,904 (23.3) | 174,993 (23.9) | 18,224 (20.4) | 140,374 (24.4) | 17,306 (22.1) |
| 4 | 226,999 (23.3) | 23,489 (21.8) | 196,477 (23.5) | 24,786 (22.3) | 166,759 (22.8) | 18,549 (20.8) | 133,866 (23.3) | 16,980 (21.7) |
| 3 | 206,060 (21.1) | 21,701 (20.1) | 178,385 (21.3) | 22,642 (20.4) | 153,828 (21.0) | 18,109 (20.3) | 121,713 (21.2) | 15,680 (20.0) |
| 2 | 167,719 (17.2) | 19,995 (18.5) | 141,810 (16.9) | 19,613 (17.7) | 126,848 (17.3) | 17,247 (19.3) | 95,929 (16.7) | 14,476 (18.5) |
| 1 | 132,225 (13.6) | 18,847 (17.5) | 110,506 (13.2) | 18,002 (16.2) | 102,819 (14.1) | 16,998 (19.0) | 75,715 (13.2) | 13,742 (17.6) |
| Unknown | 7,245 (0.7) | 140 (0.1) | 8,456 (1.0) | 143 (0.1) | 6,551 (0.9) | 125 (0.1) | 6,883 (1.2) | 94 (0.1) |
| **Region** |  |  |  |  |  |  |  |  |
| East Midlands | 88,522 (9.1) | 9,663 (9.0) | 78,258 (9.3) | 10,073 (9.1) | 63,432 (8.7) | 8,102 (9.1) | 50,931 (8.9) | 7,077 (9.0) |
| East of England | 120,141 (12.3) | 11,947 (11.1) | 103,535 (12.4) | 12,706 (11.4) | 89,752 (12.3) | 10,034 (11.2) | 71,738 (12.5) | 8,930 (11.4) |
| London | 96,225 (9.9) | 12,522 (11.6) | 76,026 (9.1) | 12,269 (11.0) | 75,410 (10.3) | 9,942 (11.1) | 55,742 (9.7) | 9,078 (11.6) |
| North East | 46,988 (4.8) | 6,184 (5.7) | 40,118 (4.8) | 6,310 (5.7) | 36,877 (5.0) | 5,490 (6.2) | 27,950 (4.9) | 4,556 (5.8) |
| North West | 126,206 (12.9) | 17,051 (15.8) | 108,039 (12.9) | 17,260 (15.5) | 94,347 (12.9) | 14,400 (16.1) | 72,539 (12.6) | 12,523 (16.0) |
| South East | 166,734 (17.1) | 16,969 (15.7) | 141,763 (16.9) | 17,869 (16.1) | 125,394 (17.1) | 13,920 (15.6) | 98,006 (17.1) | 12,283 (15.7) |
| South West | 121,560 (12.5) | 10,357 (9.6) | 106,476 (12.7) | 11,273 (10.1) | 88,451 (12.1) | 8,122 (9.1) | 71,788 (12.5) | 7,351 (9.4) |
| West Midlands | 107,087 (11.0) | 12,151 (11.3) | 92,580 (11.1) | 12,315 (11.1) | 78,990 (10.8) | 10,132 (11.4) | 62,647 (10.9) | 8,648 (11.0) |
| Yorkshire and Humber | 94,699 (9.7) | 10,926 (10.1) | 81,823 (9.8) | 10,872 (9.8) | 72,594 (9.9) | 8,985 (10.1) | 56,256 (9.8) | 7,738 (9.9) |
| Unknown | 7,245 (0.7) | 140 (0.1) | 8,456 (1.0) | 143 (0.1) | 6,551 (0.9) | 125 (0.1) | 6,883 (1.2) | 94 (0.1) |
| **Health Characteristics** |  |  |  |  |  |  |  |  |
| **Atrial fibrillation (QOF)** | 72,439 (7.4) | 10,232 (9.5) | 104,276 (12.5) | 16,598 (14.9) | 87,670 (12.0) | 13,485 (15.1) | 99,822 (17.4) | 16,234 (20.7) |
| **Asthma (QOF)** | 89,064 (9.1) | 12,552 (11.6) | 54,463 (6.5) | 9,054 (8.2) | 65,908 (9.0) | 9,992 (11.2) | 38,333 (6.7) | 6,409 (8.2) |
| **Cancer (QOF)** | 113,452 (11.6) | 13,207 (12.2) | 131,589 (15.7) | 19,357 (17.4) | 88,347 (12.1) | 11,196 (12.5) | 106,486 (18.5) | 15,175 (19.4) |
| **COPD (QOF)** | 75,089 (7.7) | 11,125 (10.3) | 83,047 (9.9) | 13,635 (12.3) | 55,335 (7.6) | 9,428 (10.6) | 60,190 (10.5) | 10,260 (13.1) |
| **Heat failure (QOF)** | 27,890 (2.9) | 4,646 (4.3) | 42,322 (5.1) | 7,410 (6.7) | 36,462 (5.0) | 6,450 (7.2) | 42,747 (7.4) | 7,696 (9.8) |
| **Palliative care (QOF)** | 13,622 (1.4) | 3,207 (3.0) | 13,029 (1.6) | 2,710 (2.4) | 17,534 (2.4) | 5,074 (5.7) | 14,337 (2.5) | 3,304 (4.2) |
| **Bipolar & schizophrenia (GPES)** | 7,714 (0.8) | 1,779 (1.6) | 4,811 (0.6) | 1,189 (1.1) | 4,968 (0.7) | 1,364 (1.5) | 2,555 (0.4) | 688 (0.9) |
| **Coronary heart disease (GPES)** | 85,148 (8.7) | 12,446 (11.5) | 158,283 (18.9) | 24,436 (22.0) | 88,767 (12.1) | 13,943 (15.6) | 133,580 (23.3) | 21,023 (26.9) |
| **Epilepsy (GPES)** | 12,835 (1.3) | 2,105 (2.0) | 12,101 (1.4) | 2,166 (1.9) | 8,910 (1.2) | 1,769 (2.0) | 8,273 (1.4) | 1,538 (2.0) |
| **Learning disability & Downs (GPES)** | 1,377 (0.1) | 603 (0.6) | 1,541 (0.2) | 662 (0.6) | 655 (0.1) | 295 (0.3) | 596 (0.1) | 311 (0.4) |
| **Liver cirrhosis (GPES)** | 4,479 (0.5) | 720 (0.7) | 3,317 (0.4) | 628 (0.6) | 2,987 (0.4) | 526 (0.6) | 1,842 (0.3) | 347 (0.4) |
| **Stroke and TIA (GPES)** | 60,647 (6.2) | 9,281 (8.6) | 73,368 (8.8) | 12,283 (11.1) | 66,639 (9.1) | 11,335 (12.7) | 67,971 (11.8) | 11,636 (14.9) |
| **Chronic respiratory disease (HES)** | 130,694 (13.4) | 21,129 (19.6) | 106,239 (12.7) | 19,661 (17.7) | 106,949 (14.6) | 19,290 (21.6) | 83,049 (14.5) | 15,933 (20.4) |
| **Cardiovascular disease (HES)** | 402,203 (41.2) | 55,361 (51.3) | 393,247 (47.0) | 62,727 (56.5) | 377,001 (51.5) | 56,931 (63.8) | 319,602 (55.6) | 52,300 (66.8) |
| **Chronic kidney disease** |  |  |  |  |  |  |  |  |
| No chronic kidney disease | 805,834 (82.6) | 85,448 (79.2) | 701,468 (83.8) | 89,586 (80.6) | 540,273 (73.8) | 62,251 (69.7) | 433,915 (75.5) | 56,085 (71.6) |
| Chronic kidney disease (QOF only) | 112,822 (11.6) | 12,395 (11.5) | 81,175 (9.7) | 10,577 (9.5) | 120,854 (16.5) | 13,509 (15.1) | 79,310 (13.8) | 9,929 (12.7) |
| Chronic kidney disease (HES) | 56,751 (5.8) | 10,067 (9.3) | 54,431 (6.5) | 10,927 (9.8) | 70,671 (9.7) | 13,492 (15.1) | 61,255 (10.7) | 12,264 (15.7) |
| **Dementia (QOF or HES)** | 31,742 (3.3) | 11,245 (10.4) | 25,365 (3.0) | 8,816 (7.9) | 50,618 (6.9) | 18,952 (21.2) | 35,332 (6.2) | 12,257 (15.7) |
| **Diabetes** |  |  |  |  |  |  |  |  |
| No diabetes | 821,177 (84.2) | 85,778 (79.5) | 655,442 (78.3) | 82,210 (74.0) | 598,200 (81.7) | 67,968 (76.2) | 439,096 (76.4) | 55,600 (71.0) |
| Diabetes (QOF only) | 55,750 (5.7) | 5,647 (5.2) | 63,893 (7.6) | 7,444 (6.7) | 42,481 (5.8) | 4,346 (4.9) | 40,612 (7.1) | 4,716 (6.0) |
| Diabetes (HES) | 98,480 (10.1) | 16,485 (15.3) | 117,739 (14.1) | 21,436 (19.3) | 91,117 (12.5) | 16,938 (19.0) | 94,772 (16.5) | 17,962 (22.9) |
| **Clinically Vulnerable (NIMS** | 137,373 (14.1) | 27,272 (25.3) | 176,344 (21.1) | 33,466 (30.1) | 136,074 (18.6) | 33,020 (37.0) | 186,751 (32.5) | 33,543 (42.9) |
| **Care home (GPES)** | 7,045 (0.7) | 5,701 (5.3) | 5,132 (0.6) | 4,087 (3.7) | 12,964 (1.8) | 10,007 (11.2) | 7,417 (1.3) | 5,321 (6.8) |
| **Smoking status (NHSD)** |  |  |  |  |  |  |  |  |
| Current Smoker | 60,541 (6.2) | 5,361 (5.0) | 59,360 (7.1) | 5,852 (5.3) | 30,899 (4.2) | 3,604 (4.0) | 28,066 (4.9) | 3,309 (4.2) |
| Ex-Smoker | 275,367 (28.2) | 34,164 (31.7) | 370,544 (44.3) | 52,494 (47.3) | 181,029 (24.7) | 24,670 (27.6) | 252,441 (43.9) | 35,694 (45.6) |
| Never Smoked | 526,777 (54.0) | 56,353 (52.2) | 309,137 (36.9) | 40,717 (36.7) | 436,708 (59.7) | 50,537 (56.6) | 225,055 (39.2) | 29,980 (38.3) |
| Non-Smoker | 26,784 (2.7) | 3,173 (2.9) | 20,287 (2.4) | 3,002 (2.7) | 20,539 (2.8) | 2,752 (3.1) | 14,348 (2.5) | 2,166 (2.8) |
| No record | 85,938 (8.8) | 8,859 (8.2) | 77,746 (9.3) | 9,025 (8.1) | 62,623 (8.6) | 7,689 (8.6) | 54,570 (9.5) | 7,129 (9.1) |
| **1st Vaccination** | 910,495 (93.3) | 98,463 (91.2) | 769,128 (91.9) | 99,043 (89.2) | 667,009 (91.1) | 77,121 (86.4) | 512,744 (89.3) | 64,477 (82.4) |
| **2nd Vaccination** | 900,327 (92.3) | 96,407 (89.3) | 758,991 (90.7) | 96,906 (87.2) | 657,046 (89.8) | 74,323 (83.3) | 503,440 (87.6) | 61,803 (79.0) |
| **Booster Vaccination** | 857,460 (87.9) | 89,308 (82.8) | 721,510 (86.2) | 90,476 (81.4) | 613,386 (83.8) | 66,155 (74.1) | 467,244 (81.3) | 55,215 (70.5) |
| **Deaths*** | 66,088 (6.8) | 15,925 (14.8) | 80,185 (9.6) | 21,125 (19.0) | 89,233 (12.2) | 23,685 (26.5) | 93,180 (16.2) | 25,995 (33.2) |
| **Survival times (median[IQR])** | 130 [130.0, 130.00] | 130 [130.0, 130.00] | 130 [130.0, 130.00] | 130 [130.0, 130.00] | 130 [130.0, 130.00] | 130 [122.0, 130.00] | 130 [130.0, 130.00] | 130 [99.0, 130.00] |
|  |  |  |  |  |  |  |  |  |
|  | **85-89-year-olds** | | | | **90+-year-olds** | | | |
|  | **Female** | | **Male** | | **Female** | | **Male** | |
|  | No positive SARS‑CoV‑2 test | Positive SARS‑CoV‑2 test | No positive SARS‑CoV‑2 test | Positive SARS‑CoV‑2 test | No positive SARS‑CoV‑2 test | Positive SARS‑CoV‑2 test | No positive SARS‑CoV‑2 test | Positive SARS‑CoV‑2 test |
| **n** | 459,172·0 | 74,537·0 | 310,933 | 50,028·0 | 286,484·0 | 64,073·0 | 138,608·0 | 27,183 |
| **Age (mean(SD)** | 86·9 (1·40) | 86·9 (1·41) | 87 (1·39) | 87 (1·40) | 93 (2·84) | 93 (2·85) | 92 (2·47) | 93 (2·47) |
| **Ethnicity** |  |  |  |  |  |  |  |  |
| Asian | 12,478 (2·7) | 2,056 (2·8) | 10,644 (3·4) | 2,035 (4·1) | 5,671 (2·0) | 947 (1·5) | 3,856 (2·8) | 739 (2·7) |
| Black | 7,085 (1·5) | 1,231 (1·7) | 5,407 (1·7) | 1,073 (2·1) | 3,203 (1·1) | 609 (1·0) | 2,058 (1·5) | 457 (1·7) |
| Mixed | 1,500 (0·3) | 199 (0·3) | 919 (0·3) | 153 (0·3) | 843 (0·3) | 154 (0·2) | 378 (0·3) | 92 (0·3) |
| Other | 1,637 (0·4) | 160 (0·2) | 1,036 (0·3) | 127 (0·3) | 933 (0·3) | 141 (0·2) | 445 (0·3) | 70 (0·3) |
| White | 423,398 (92·2) | 70,274 (94·3) | 282,548 (90·9) | 46,232 (92·4) | 264,877 (92·5) | 61,745 (96·4) | 124,650 (89·9) | 25,619 (94·2) |
| Unknown | 13,074 (2·8) | 617 (0·8) | 10,379 (3·3) | 408 (0·8) | 10,957 (3·8) | 477 (0·7) | 7,221 (5·2) | 206 (0·8) |
| **IMD Quintile** |  |  |  |  |  |  |  |  |
| 5 (least deprived) | 109,422 (23·8) | 15,029 (20·2) | 78,629 (25·3) | 10,985 (22·0) | 66,644 (23·3) | 13,958 (21·8) | 34,606 (25·0) | 6,292 (23·1) |
| 4 | 104,237 (22·7) | 15,564 (20·9) | 72,842 (23·4) | 11,090 (22·2) | 64,690 (22·6) | 13,911 (21·7) | 31,832 (23·0) | 6,048 (22·2) |
| 3 | 97,354 (21·2) | 15,302 (20·5) | 65,179 (21·0) | 10,089 (20·2) | 60,936 (21·3) | 13,179 (20·6) | 28,895 (20·8) | 5,613 (20·6) |
| 2 | 79,373 (17·3) | 14,664 (19·7) | 50,922 (16·4) | 9,239 (18·5) | 49,811 (17·4) | 12,257 (19·1) | 22,539 (16·3) | 4,901 (18·0) |
| 1 | 63,447 (13·8) | 13,878 (18·6) | 38,544 (12·4) | 8,551 (17·1) | 38,645 (13·5) | 10,688 (16·7) | 16,990 (12·3) | 4,293 (15·8) |
| Unknown | 5,339 (1·2) | 100 (0·1) | 4,817 (1·5) | 74 (0·1) | 5,758 (2·0) | 80 (0·1) | 3,746 (2·7) | 36 (0·1) |
| **Region** |  |  |  |  |  |  |  |  |
| East Midlands | 38,589 (8·4) | 6,903 (9·3) | 26,774 (8·6) | 4,694 (9·4) | 23,456 (8·2) | 6,072 (9·5) | 11,325 (8·2) | 2,513 (9·2) |
| East of England | 57,776 (12·6) | 8,576 (11·5) | 39,837 (12·8) | 6,020 (12·0) | 36,323 (12·7) | 7,926 (12·4) | 17,787 (12·8) | 3,358 (12·4) |
| London | 46,331 (10·1) | 7,536 (10·1) | 30,880 (9·9) | 5,687 (11·4) | 29,479 (10·3) | 5,940 (9·3) | 14,074 (10·2) | 2,986 (11·0) |
| North East | 22,087 (4·8) | 4,548 (6·1) | 14,510 (4·7) | 2,813 (5·6) | 12,013 (4·2) | 3,516 (5·5) | 5,735 (4·1) | 1,330 (4·9) |
| North West | 55,879 (12·2) | 11,440 (15·3) | 36,673 (11·8) | 7,499 (15·0) | 32,185 (11·2) | 9,113 (14·2) | 14,958 (10·8) | 3,765 (13·9) |
| South East | 81,698 (17·8) | 12,437 (16·7) | 55,134 (17·7) | 8,250 (16·5) | 53,181 (18·6) | 11,571 (18·1) | 25,458 (18·4) | 4,921 (18·1) |
| South West | 58,167 (12·7) | 7,323 (9·8) | 40,221 (12·9) | 4,724 (9·4) | 38,739 (13·5) | 6,780 (10·6) | 18,723 (13·5) | 2,805 (10·3) |
| West Midlands | 48,693 (10·6) | 8,335 (11·2) | 32,622 (10·5) | 5,654 (11·3) | 29,296 (10·2) | 7,188 (11·2) | 14,409 (10·4) | 2,999 (11·0) |
| Yorkshire and Humber | 44,613 (9·7) | 7,339 (9·8) | 29,465 (9·5) | 4,613 (9·2) | 26,054 (9·1) | 5,887 (9·2) | 12,393 (8·9) | 2,470 (9·1) |
| Unknown | 5,339 (1·2) | 100 (0·1) | 4,817 (1·5) | 74 (0·1) | 5,758 (2·0) | 80 (0·1) | 3,746 (2·7) | 36 (0·1) |
| **Health Characteristics** |  |  |  |  |  |  |  |  |
| **Atrial fibrillation (QOF)** | 76,214 (16·6) | 14,690 (19·7) | 69,124 (22·2) | 12,759 (25·5) | 54,370 (19·0) | 13,387 (20·9) | 33,112 (23·9) | 7,423 (27·3) |
| **Asthma (QOF)** | 36,453 (7·9) | 6,732 (9·0) | 18,989 (6·1) | 3,574 (7·1) | 17,561 (6·1) | 4,361 (6·8) | 6,877 (5·0) | 1,547 (5·7) |
| **Cancer (QOF)** | 54,415 (11·9) | 8,642 (11·6) | 59,991 (19·3) | 9,815 (19·6) | 30,374 (10·6) | 6,602 (10·3) | 24,324 (17·5) | 4,810 (17·7) |
| **COPD (QOF)** | 30,814 (6·7) | 6,259 (8·4) | 30,394 (9·8) | 6,005 (12·0) | 15,055 (5·3) | 3,955 (6·2) | 11,765 (8·5) | 2,699 (9·9) |
| **Heat failure (QOF)** | 34,510 (7·5) | 7,104 (9·5) | 31,919 (10·3) | 6,341 (12·7) | 28,226 (9·9) | 7,035 (11·0) | 16,740 (12·1) | 3,706 (13·6) |
| **Palliative care (QOF)** | 20,148 (4·4) | 6,780 (9·1) | 12,349 (4·0) | 3,226 (6·4) | 30,492 (10·6) | 10,137 (15·8) | 10,880 (7·8) | 2,842 (10·5) |
| **Bipolar & schizophrenia (GPES)** | 2,657 (0·6) | 904 (1·2) | 996 (0·3) | 293 (0·6) | 1,364 (0·5) | 473 (0·7) | 335 (0·2) | 120 (0·4) |
| **Coronary heart disease (GPES)** | 69,446 (15·1) | 13,315 (17·9) | 81,784 (26·3) | 14,304 (28·6) | 48,335 (16·9) | 12,061 (18·8) | 36,222 (26·1) | 7,810 (28·7) |
| **Epilepsy (GPES)** | 5,323 (1·2) | 1,355 (1·8) | 4,295 (1·4) | 943 (1·9) | 2,922 (1·0) | 831 (1·3) | 1,692 (1·2) | 457 (1·7) |
| **Learning disability & Downs (GPES)** | 293 (0·1) | 132 (0·2) | 234 (0·1) | 117 (0·2) | 102 (0·0) | 47 (0·1) | 61 (0·0) | 36 (0·1) |
| **Liver cirrhosis (GPES)** | 1,435 (0·3) | 288 (0·4) | 756 (0·2) | 150 (0·3) | 533 (0·2) | 135 (0·2) | 195 (0·1) | 45 (0·2) |
| **Stroke and TIA (GPES)** | 56,226 (12·2) | 11,928 (16·0) | 45,318 (14·6) | 8,957 (17·9) | 42,962 (15·0) | 11,521 (18·0) | 22,770 (16·4) | 5,088 (18·7) |
| **Chronic respiratory disease (HES)** | 65,705 (14·3) | 14,458 (19·4) | 46,370 (14·9) | 10,074 (20·1) | 36,190 (12·6) | 10,204 (15·9) | 19,379 (14·0) | 4,821 (17·7) |
| **Cardiovascular disease (HES)** | 272,518 (59·3) | 53,167 (71·3) | 194,370 (62·5) | 36,804 (73·6) | 182,829 (63·8) | 47,166 (73·6) | 90,295 (65·1) | 20,673 (76·1) |
| **Chronic kidney disease** |  |  |  |  |  |  |  |  |
| No chronic kidney disease | 299,644 (65·3) | 46,637 (62·6) | 207,470 (66·7) | 31,545 (63·1) | 168,891 (59·0) | 36,574 (57·1) | 83,458 (60·2) | 15,589 (57·3) |
| Chronic kidney disease (QOF only) | 95,244 (20·7) | 13,211 (17·7) | 54,310 (17·5) | 7,555 (15·1) | 65,987 (23·0) | 12,858 (20·1) | 26,711 (19·3) | 4,516 (16·6) |
| Chronic kidney disease (HES) | 64,284 (14·0) | 14,689 (19·7) | 49,153 (15·8) | 10,928 (21·8) | 51,606 (18·0) | 14,641 (22·9) | 28,439 (20·5) | 7,078 (26·0) |
| **Dementia (QOF or HES)** | 58,887 (12·8) | 24,044 (32·3) | 32,162 (10·3) | 11,543 (23·1) | 60,985 (21·3) | 23,959 (37·4) | 19,900 (14·4) | 6,918 (25·4) |
| **Diabetes** |  |  |  |  |  |  |  |  |
| No diabetes | 375,515 (81·8) | 58,079 (77·9) | 238,890 (76·8) | 36,043 (72·0) | 243,188 (84·9) | 53,234 (83·1) | 112,226 (81·0) | 21,113 (77·7) |
| Diabetes (QOF only) | 24,161 (5·3) | 3,031 (4·1) | 19,003 (6·1) | 2,508 (5·0) | 11,763 (4·1) | 2,021 (3·2) | 6,337 (4·6) | 1,015 (3·7) |
| Diabetes (HES) | 59,496 (13·0) | 13,427 (18·0) | 53,040 (17·1) | 11,477 (22·9) | 31,533 (11·0) | 8,818 (13·8) | 20,045 (14·5) | 5,055 (18·6) |
| **Clinically Vulnerable (NIMS** | 118,228 (25·7) | 36,154 (48·5) | 134,502 (43·3) | 26,161 (52·3) | 97,209 (33·9) | 35,225 (55·0) | 75,067 (54·2) | 15,546 (57·2) |
| **Care home (GPES)** | 19,114 (4·2) | 13,739 (18·4) | 8,392 (2·7) | 5,416 (10·8) | 28,501 (9·9) | 16,402 (25·6) | 7,897 (5·7) | 4,342 (16·0) |
| **Smoking status (NHSD)** |  |  |  |  |  |  |  |  |
| Current Smoker | 12,484 (2·7) | 2,043 (2·7) | 9,562 (3·1) | 1,382 (2·8) | 4,260 (1·5) | 879 (1·4) | 2,906 (2·1) | 556 (2·0) |
| Ex-Smoker | 102,802 (22·4) | 17,986 (24·1) | 130,197 (41·9) | 21,777 (43·5) | 59,426 (20·7) | 14,045 (21·9) | 57,532 (41·5) | 11,780 (43·3) |
| Never Smoked | 289,527 (63·1) | 45,230 (60·7) | 131,100 (42·2) | 20,248 (40·5) | 180,371 (63·0) | 41,022 (64·0) | 55,225 (39·8) | 11,039 (40·6) |
| Non-Smoker | 13,292 (2·9) | 2,518 (3·4) | 7,967 (2·6) | 1,502 (3·0) | 8,982 (3·1) | 2,371 (3·7) | 3,563 (2·6) | 863 (3·2) |
| No record | 41,067 (8·9) | 6,760 (9·1) | 32,107 (10·3) | 5,119 (10·2) | 33,445 (11·7) | 5,756 (9·0) | 19,382 (14·0) | 2,945 (10·8) |
| **1st Vaccination** | 397,309 (86·5) | 61,230 (82·1) | 261,000 (83·9) | 37,509 (75·0) | 212,214 (74·1) | 49,243 (76·9) | 98,847 (71·3) | 18,152 (66·8) |
| **2nd Vaccination** | 387,297 (84·3) | 57,803 (77·5) | 253,529 (81·5) | 34,886 (69·7) | 201,270 (70·3) | 45,212 (70·6) | 93,394 (67·4) | 16,264 (59·8) |
| **Booster Vaccination** | 350,252 (76·3) | 49,676 (66·6) | 226,496 (72·8) | 29,591 (59·1) | 168,597 (58·9) | 37,375 (58·3) | 77,352 (55·8) | 13,025 (47·9) |
| **Deaths*** | 102,659 (22·4) | 28,687 (38·5) | 84,835 (27·3) | 24,646 (49·3) | 124,798 (43·6) | 33,368 (52·1) | 63,886 (46·1) | 17,579 (64·7) |
| **Survival times (median[IQR])** | 130 [125·0, 130·00] | 130 [90·0, 130·00] | 130 [109·0, 130·00] | 130 [50·0, 130·00] | 130 [60·0, 130·00] | 124 [54·0, 130·00] | 130 [55·0, 130·00] | 99 [45·0, 130·00] |

Data are presented as n (%) unless otherwise specified. 1st positive COVID-19 tests from 1 March 2020 to 31 March 2022. *Deaths are defined according to censor
