# Supplementary material for: Risk of death in England following a positive SARS-CoV-2 test: A retrospective national cohort study (March 2020 to September 2022)
Source: PLoS One. 2024 Oct 9;19(10):e0304110. doi: 10.1371/journal.pone.0304110 (PMC11463829; doi:10.1371/journal.pone.0304110)
Supplement: S2 Table — (DOCX) [file pone.0304110.s009.docx]

**Table S2. Hazard ratios and 95% confidence intervals derived from the Cox Proportional Hazard Model denoting the hazard of deaths stratified by age group and sex**

|  |  | **45-49 years** | |  | **50-54 years** | |  | **55-59 years** | |
| --- | --- | --- | --- | --- | --- | --- | --- | --- | --- |
|  |  | **Female** | **Male** |  | **Female** | **Male** |  | **Female** | **Male** |
| **Age** | | 1·06 (1·04 to 1·07) | 1·08 (1·06 to 1·09) |  | 1·06 (1·04 to 1·07) | 1·06 (1·05 to 1·07) |  | 1·05 (1·04 to 1·06) | 1·07 (1·06 to 1·07) |
| **Ethnicity** | |  |  |  |  |  |  |  |  |
|  | White ^ref^ |  |  |  |  |  |  |  |  |
|  | Black | 0·91 (0·82 to 1·02) | 0·74 (0·68 to 0·82) |  | 0·97 (0·89 to 1·05) | 0·75 (0·7 to 0·81) |  | 0·94 (0·87 to 1·01) | 0·82 (0·77 to 0·86) |
|  | Asian | 0·7 (0·64 to 0·77) | 0·61 (0·57 to 0·66) |  | 0·72 (0·67 to 0·78) | 0·66 (0·62 to 0·7) |  | 0·73 (0·68 to 0·79) | 0·75 (0·71 to 0·79) |
|  | Mixed | 0·86 (0·71 to 1·04) | 0·59 (0·49 to 0·7) |  | 0·8 (0·67 to 0·95) | 0·67 (0·58 to 0·78) |  | 0·77 (0·66 to 0·9) | 0·77 (0·68 to 0·87) |
|  | Other | 0·54 (0·42 to 0·69) | 0·45 (0·38 to 0·54) |  | 0·68 (0·56 to 0·83) | 0·6 (0·53 to 0·69) |  | 0·52 (0·43 to 0·64) | 0·66 (0·58 to 0·75) |
|  | Unknown | 0·96 (0·85 to 1·08) | 0·67 (0·62 to 0·72) |  | 1·11 (1·01 to 1·22) | 0·76 (0·71 to 0·8) |  | 1·3 (1·21 to 1·4) | 0·87 (0·83 to 0·92) |
| **IMD Quintile** | |  |  |  |  |  |  |  |  |
|  | Q5 (least deprived)  ^ref^ |  |  |  |  |  |  |  |  |
|  | Q4 | 1·14 (1·06 to 1·24) | 1·17 (1·09 to 1·25) |  | 1·08 (1·01 to 1·15) | 1·11 (1·05 to 1·17) |  | 1·1 (1·05 to 1·16) | 1·11 (1·06 to 1·16) |
|  | Q3 | 1·3 (1·2 to 1·4) | 1·38 (1·29 to 1·47) |  | 1·19 (1·12 to 1·26) | 1·24 (1·18 to 1·3) |  | 1·19 (1·13 to 1·25) | 1·23 (1·18 to 1·28) |
|  | Q2 | 1·47 (1·36 to 1·58) | 1·57 (1·47 to 1·67) |  | 1·31 (1·24 to 1·39) | 1·44 (1·37 to 1·51) |  | 1·3 (1·24 to 1·37) | 1·39 (1·33 to 1·45) |
|  | Q1 (most deprived) | 1·64 (1·52 to 1·77) | 1·93 (1·81 to 2·05) |  | 1·47 (1·39 to 1·56) | 1·69 (1·61 to 1·77) |  | 1·42 (1·35 to 1·49) | 1·61 (1·55 to 1·68) |
|  | Unknown | 4·21 (3·54 to 5) | 3·84 (3·37 to 4·39) |  | 4·48 (3·9 to 5·15) | 2·92 (2·62 to 3·26) |  | 4·71 (4·21 to 5·27) | 2·82 (2·58 to 3·08) |
| **Region** | |  |  |  |  |  |  |  |  |
|  | London ^ref^ |  |  |  |  |  |  |  |  |
|  | East Midlands | 1·27 (1·16 to 1·4) | 1·33 (1·24 to 1·44) |  | 1·2 (1·12 to 1·3) | 1·17 (1·1 to 1·24) |  | 1·18 (1·11 to 1·26) | 1·08 (1·03 to 1·13) |
|  | East of England | 1·05 (0·96 to 1·15) | 1·18 (1·09 to 1·27) |  | 1·16 (1·08 to 1·25) | 1·12 (1·06 to 1·19) |  | 1·07 (1·01 to 1·14) | 1·05 (1 to 1·1) |
|  | North East | 1·22 (1·1 to 1·36) | 1·26 (1·15 to 1·37) |  | 1·15 (1·06 to 1·26) | 1·17 (1·09 to 1·26) |  | 1·14 (1·07 to 1·23) | 1·04 (0·98 to 1·1) |
|  | North West | 1·28 (1·18 to 1·38) | 1·29 (1·21 to 1·38) |  | 1·17 (1·09 to 1·25) | 1·18 (1·12 to 1·25) |  | 1·13 (1·07 to 1·2) | 1·06 (1·02 to 1·11) |
|  | South East | 1·1 (1·01 to 1·2) | 1·19 (1·11 to 1·27) |  | 1·2 (1·12 to 1·28) | 1·12 (1·06 to 1·18) |  | 1·15 (1·08 to 1·21) | 1·05 (1·01 to 1·1) |
|  | South West | 1·14 (1·04 to 1·25) | 1·17 (1·09 to 1·27) |  | 1·15 (1·06 to 1·24) | 1·12 (1·05 to 1·18) |  | 1·07 (1·01 to 1·14) | 1 (0·95 to 1·05) |
|  | West Midlands | 1·26 (1·16 to 1·38) | 1·35 (1·26 to 1·44) |  | 1·19 (1·11 to 1·28) | 1·18 (1·12 to 1·25) |  | 1·12 (1·05 to 1·18) | 1·1 (1·05 to 1·16) |
|  | Yorkshire Humber | 1·27 (1·16 to 1·39) | 1·34 (1·25 to 1·44) |  | 1·2 (1·11 to 1·29) | 1·2 (1·13 to 1·27) |  | 1·14 (1·08 to 1·22) | 1·07 (1·02 to 1·13) |
| **Health characteristics** | |  |  |  |  |  |  |  |  |
|  | Atrial fibrillation (QOF) | 1·29 (1·03 to 1·62) | 1·26 (1·11 to 1·44) |  | 1·17 (1·01 to 1·36) | 1·22 (1·12 to 1·33) |  | 1·36 (1·24 to 1·49) | 1·16 (1·09 to 1·23) |
|  | Asthma (QOF) | 0·89 (0·83 to 0·96) | 0·94 (0·87 to 1) |  | 0·85 (0·81 to 0·9) | 0·83 (0·78 to 0·88) |  | 0·84 (0·8 to 0·88) | 0·84 (0·81 to 0·88) |
|  | Cancer (QOF) | 6·13 (5·78 to 6·5) | 4·64 (4·34 to 4·96) |  | 4·73 (4·53 to 4·95) | 3·94 (3·76 to 4·14) |  | 3·64 (3·51 to 3·78) | 3·48 (3·36 to 3·6) |
|  | COPD (QOF) | 1·4 (1·26 to 1·55) | 1·43 (1·3 to 1·57) |  | 1·32 (1·23 to 1·42) | 1·31 (1·23 to 1·39) |  | 1·31 (1·24 to 1·38) | 1·34 (1·28 to 1·4) |
|  | Heart failure (QOF) | 1·85 (1·56 to 2·18) | 1·54 (1·36 to 1·74) |  | 1·63 (1·45 to 1·83) | 1·91 (1·77 to 2·07) |  | 1·62 (1·49 to 1·77) | 1·64 (1·54 to 1·73) |
|  | Palliative care (QOF) | 7·05 (6·48 to 7·68) | 6·03 (5·46 to 6·67) |  | 7·75 (7·28 to 8·26) | 5·7 (5·31 to 6·12) |  | 6·97 (6·61 to 7·34) | 5·14 (4·88 to 5·42) |
|  | Bipolar & schizophrenia (GPES) | 1·4 (1·24 to 1·59) | 1·98 (1·82 to 2·16) |  | 1·53 (1·39 to 1·68) | 1·76 (1·63 to 1·89) |  | 1·52 (1·41 to 1·65) | 1·65 (1·55 to 1·76) |
|  | Coronary heart disease (GPES) | 0·94 (0·81 to 1·1) | 0·87 (0·8 to 0·96) |  | 0·98 (0·89 to 1·08) | 0·9 (0·84 to 0·95) |  | 0·96 (0·9 to 1·02) | 0·92 (0·89 to 0·96) |
|  | Epilepsy (GPES) | 1·65 (1·49 to 1·83) | 1·98 (1·83 to 2·15) |  | 1·49 (1·37 to 1·62) | 1·72 (1·61 to 1·83) |  | 1·38 (1·29 to 1·48) | 1·53 (1·45 to 1·62) |
|  | Learning dis· & Downs (GPES) | 3·05 (2·68 to 3·47) | 1·89 (1·69 to 2·12) |  | 2·69 (2·42 to 3) | 2·31 (2·12 to 2·51) |  | 2·99 (2·74 to 3·27) | 2·21 (2·05 to 2·38) |
|  | Liver cirrhosis (GPES) | 5·7 (5·07 to 6·42) | 4·58 (4·16 to 5·03) |  | 4·16 (3·77 to 4·59) | 4·24 (3·95 to 4·56) |  | 3·46 (3·19 to 3·76) | 3·02 (2·84 to 3·22) |
|  | Stroke & TIA (GPES) | 1·32 (1·14 to 1·52) | 1·2 (1·07 to 1·34) |  | 1·24 (1·13 to 1·37) | 1·29 (1·2 to 1·39) |  | 1·29 (1·2 to 1·38) | 1·27 (1·2 to 1·33) |
|  | Chronic respiratory disease (HES) | 1·5 (1·4 to 1·62) | 1·56 (1·46 to 1·66) |  | 1·48 (1·4 to 1·56) | 1·55 (1·47 to 1·63) |  | 1·35 (1·29 to 1·41) | 1·44 (1·38 to 1·5) |
|  | Cardiovascular disease (HES) | 2·58 (2·44 to 2·72) | 2·73 (2·6 to 2·86) |  | 2·22 (2·13 to 2·32) | 2·23 (2·15 to 2·32) |  | 2·05 (1·98 to 2·12) | 1·91 (1·86 to 1·97) |
|  | Chronic kidney disease (QOF only) | 1·74 (1·43 to 2·11) | 1·58 (1·3 to 1·92) |  | 1·33 (1·17 to 1·51) | 1·33 (1·17 to 1·51) |  | 1·27 (1·16 to 1·38) | 1·3 (1·19 to 1·41) |
|  | Chronic kidney disease (HES) | 1·95 (1·74 to 2·18) | 2·26 (2·06 to 2·48) |  | 2·36 (2·18 to 2·55) | 2·06 (1·92 to 2·2) |  | 1·99 (1·87 to 2·12) | 2·14 (2·04 to 2·26) |
|  | Dementia (HES or QOF) | 3·15 (2·46 to 4·04) | 2·8 (2·23 to 3·51) |  | 3·25 (2·79 to 3·77) | 2·37 (2·06 to 2·73) |  | 2·79 (2·52 to 3·1) | 2·14 (1·95 to 2·35) |
|  | Diabetes (QOF only) | 1·07 (0·94 to 1·22) | 1·39 (1·27 to 1·52) |  | 1·18 (1·08 to 1·3) | 1·29 (1·22 to 1·38) |  | 1·11 (1·03 to 1·19) | 1·31 (1·25 to 1·38) |
|  | Diabetes (HES) | 1·29 (1·19 to 1·39) | 1·63 (1·53 to 1·74) |  | 1·35 (1·27 to 1·43) | 1·5 (1·43 to 1·57) |  | 1·38 (1·32 to 1·44) | 1·44 (1·39 to 1·49) |
|  | Clinically vulnerable (NIMS) | 2·26 (2·13 to 2·4) | 1·53 (1·45 to 1·62) |  | 2·19 (2·1 to 2·3) | 1·59 (1·53 to 1·66) |  | 2·11 (2·04 to 2·19) | 1·57 (1·52 to 1·62) |
|  | Care home resident (GPES) | 3·83 (3 to 4·89) | 1·43 (1·13 to 1·81) |  | 3·67 (3·09 to 4·36) | 1·59 (1·35 to 1·86) |  | 2 (1·74 to 2·29) | 2·39 (2·15 to 2·65) |
| **Smoking status** | |  |  |  |  |  |  |  |  |
|  | Never smoker (NHSD) ^ref^ |  |  |  |  |  |  |  |  |
|  | Current smoker (NHSD) | 2·16 (2·04 to 2·29) | 2·54 (2·43 to 2·66) |  | 2·11 (2·02 to 2·21) | 2·47 (2·39 to 2·57) |  | 2·17 (2·09 to 2·25) | 2·4 (2·33 to 2·47) |
|  | Ex-smoker (NHSD) | 1·19 (1·12 to 1·26) | 1·21 (1·15 to 1·28) |  | 1·19 (1·14 to 1·25) | 1·24 (1·19 to 1·29) |  | 1·32 (1·27 to 1·37) | 1·28 (1·24 to 1·32) |
|  | Non-smoker (NHSD) | 1·06 (0·93 to 1·21) | 1·28 (1·15 to 1·44) |  | 1·04 (0·93 to 1·16) | 1·13 (1·03 to 1·25) |  | 1·12 (1·02 to 1·22) | 1·12 (1·04 to 1·22) |
|  | No record (NHSD) | 2·13 (1·98 to 2·29) | 2·49 (2·36 to 2·64) |  | 2·21 (2·08 to 2·34) | 2·4 (2·29 to 2·51) |  | 2·19 (2·08 to 2·3) | 2·44 (2·35 to 2·54) |
| **Wave & COVID-19 & Vaccination status** | |  |  |  |  |  |  |  |  |
|  | Wave 1 & 0-4 weeks & unvaccinated | 51·6 (42·6 to 62·51) | 66·64 (57·22 to 77·62) |  | 58·77 (51·04 to 67·66) | 79·76 (71·42 to 89·08) |  | 57·93 (51·5 to 65·16) | 88·69 (81·82 to 96·13) |
|  | Wave 1 & 5-26 weeks & unvaccinated | 4·84 (3·66 to 6·38) | 5·52 (4·36 to 6·99) |  | 4·04 (3·19 to 5·12) | 5·29 (4·38 to 6·38) |  | 3·79 (3·1 to 4·65) | 5·75 (4·99 to 6·63) |
|  | Wave 2 & 0-4 weeks & unvaccinated | 15·81 (13·83 to 18·07) | 17·5 (15·66 to 19·56) |  | 19·35 (17·52 to 21·36) | 20·6 (18·98 to 22·35) |  | 22·61 (20·81 to 24·55) | 24·94 (23·37 to 26·61) |
|  | Wave 2 & 0-4 weeks & 1st vaccine | 7·47 (3·1 to 18·02) | 2·96 (0·42 to 21·04) |  | 9·47 (5·08 to 17·65) | 16·48 (8·85 to 30·69) |  | 13·56 (8·52 to 21·58) | 15·08 (9·48 to 23·99) |
|  | Wave 2 & 5-26 weeks & unvaccinated | 2·12 (1·82 to 2·46) | 2·82 (2·5 to 3·17) |  | 2·35 (2·08 to 2·64) | 2·61 (2·37 to 2·88) |  | 2·63 (2·38 to 2·91) | 3·25 (3·01 to 3·51) |
|  | Wave 2 & 5-26 weeks & 1st vaccine | 2·36 (1·18 to 4·73) | 1·06 (0·26 to 4·24) |  | 1·93 (1·06 to 3·48) | 4·72 (2·79 to 7·98) |  | 2·09 (1·24 to 3·54) | 2·85 (1·77 to 4·59) |
|  | Wave 3 & 0-4 weeks & unvaccinated | 14·33 (11·27 to 18·21) | 23·17 (19·7 to 27·26) |  | 23·23 (19·29 to 27·99) | 29·93 (26·05 to 34·39) |  | 25·96 (22·08 to 30·52) | 32·64 (28·61 to 37·24) |
|  | Wave 3 & 0-4 weeks & 1st vaccine | 6·67 (4·02 to 11·09) | 9·35 (6·53 to 13·4) |  | 5·69 (3·3 to 9·81) | 9·89 (6·82 to 14·34) |  | 12·7 (8·7 to 18·53) | 14·23 (10·43 to 19·41) |
|  | Wave 3 & 0-4 weeks & 2nd vaccine | 3·56 (2·89 to 4·39) | 2·95 (2·4 to 3·61) |  | 3·34 (2·75 to 4·06) | 3·91 (3·35 to 4·57) |  | 5·02 (4·34 to 5·81) | 6·11 (5·46 to 6·84) |
|  | Wave 3 & 0-4 weeks & booster vaccine | 1·4 (1 to 1·97) | 1·51 (1·07 to 2·15) |  | 1·44 (1·1 to 1·88) | 1·62 (1·26 to 2·09) |  | 1·78 (1·45 to 2·19) | 2·04 (1·7 to 2·45) |
|  | Wave 3 & 5-26 weeks & unvaccinated | 2·61 (2·03 to 3·34) | 3·46 (2·87 to 4·17) |  | 2·52 (1·98 to 3·21) | 4·24 (3·6 to 4·99) |  | 2·92 (2·36 to 3·6) | 4·52 (3·86 to 5·29) |
|  | Wave 3 & 5-26 weeks & 1st vaccine | 2·06 (1·38 to 3·08) | 1·6 (1·1 to 2·33) |  | 2·33 (1·59 to 3·43) | 2·55 (1·86 to 3·51) |  | 1·86 (1·22 to 2·82) | 2·54 (1·85 to 3·5) |
|  | Wave 3 & 5-26 weeks & 2nd vaccine | 0·91 (0·75 to 1·09) | 1 (0·86 to 1·18) |  | 1·08 (0·93 to 1·25) | 1·09 (0·96 to 1·24) |  | 1·27 (1·12 to 1·44) | 1·49 (1·35 to 1·65) |
|  | Wave 3 & 5-26 weeks & booster vaccine | 0·91 (0·75 to 1·11) | 1·05 (0·86 to 1·28) |  | 0·93 (0·8 to 1·08) | 1 (0·86 to 1·16) |  | 0·95 (0·83 to 1·08) | 1·24 (1·11 to 1·38) |
|  |  |  |  |  |  |  |  |  |  |
|  |  | **60-64 years** | |  | **65-69 years** | |  | **70-74 years** | |
|  |  | **Female** | **Male** |  | **Female** | **Male** |  | **Female** | **Male** |
| **Age** | | 1·07 (1·06 to 1·08) | 1·06 (1·05 to 1·07) |  | 1·07 (1·07 to 1·08) | 1·06 (1·06 to 1·07) |  | 1·08 (1·07 to 1·09) | 1·08 (1·07 to 1·08) |
| **Ethnicity** | |  |  |  |  |  |  |  |  |
|  | White ^ref^ |  |  |  |  |  |  |  |  |
|  | Black | 0·89 (0·82 to 0·95) | 0·79 (0·74 to 0·84) |  | 0·86 (0·79 to 0·92) | 0·76 (0·71 to 0·82) |  | 0·84 (0·78 to 0·9) | 0·81 (0·75 to 0·86) |
|  | Asian | 0·81 (0·77 to 0·86) | 0·77 (0·74 to 0·8) |  | 0·84 (0·79 to 0·88) | 0·77 (0·74 to 0·8) |  | 0·9 (0·86 to 0·94) | 0·78 (0·75 to 0·82) |
|  | Mixed | 0·81 (0·69 to 0·94) | 0·81 (0·71 to 0·91) |  | 0·74 (0·63 to 0·87) | 0·77 (0·68 to 0·87) |  | 0·78 (0·68 to 0·91) | 0·85 (0·75 to 0·96) |
|  | Other | 0·59 (0·5 to 0·7) | 0·71 (0·63 to 0·8) |  | 0·68 (0·58 to 0·79) | 0·61 (0·54 to 0·7) |  | 0·69 (0·6 to 0·79) | 0·74 (0·66 to 0·83) |
|  | Unknown | 1·31 (1·23 to 1·4) | 0·93 (0·88 to 0·97) |  | 1·49 (1·41 to 1·57) | 1·18 (1·13 to 1·23) |  | 1·62 (1·56 to 1·69) | 1·33 (1·28 to 1·38) |
| **IMD Quintile** | |  |  |  |  |  |  |  |  |
|  | Q5 (least deprived)  ^ref^ |  |  |  |  |  |  |  |  |
|  | Q4 | 1·09 (1·04 to 1·13) | 1·08 (1·04 to 1·12) |  | 1·06 (1·02 to 1·09) | 1·1 (1·07 to 1·13) |  | 1·08 (1·05 to 1·11) | 1·1 (1·08 to 1·13) |
|  | Q3 | 1·19 (1·14 to 1·24) | 1·2 (1·16 to 1·24) |  | 1·13 (1·09 to 1·17) | 1·21 (1·18 to 1·25) |  | 1·15 (1·12 to 1·18) | 1·19 (1·17 to 1·22) |
|  | Q2 | 1·29 (1·24 to 1·35) | 1·35 (1·31 to 1·4) |  | 1·24 (1·2 to 1·29) | 1·34 (1·3 to 1·38) |  | 1·25 (1·22 to 1·29) | 1·31 (1·28 to 1·34) |
|  | Q1 (most deprived) | 1·42 (1·36 to 1·48) | 1·54 (1·49 to 1·59) |  | 1·36 (1·31 to 1·41) | 1·54 (1·49 to 1·58) |  | 1·39 (1·35 to 1·43) | 1·45 (1·42 to 1·49) |
|  | Unknown | 4·53 (4·11 to 4·99) | 2·64 (2·43 to 2·86) |  | 4·48 (4·12 to 4·87) | 3·25 (3·03 to 3·48) |  | 5·41 (5·07 to 5·78) | 3·97 (3·75 to 4·2) |
| **Region** | |  |  |  |  |  |  |  |  |
|  | London ^ref^ |  |  |  |  |  |  |  |  |
|  | East Midlands | 1·13 (1·07 to 1·19) | 1·12 (1·07 to 1·17) |  | 1·14 (1·09 to 1·2) | 1·09 (1·05 to 1·14) |  | 1·16 (1·12 to 1·2) | 1·09 (1·06 to 1·13) |
|  | East of England | 1·08 (1·03 to 1·14) | 1·04 (1 to 1·09) |  | 1·11 (1·06 to 1·16) | 1·07 (1·04 to 1·11) |  | 1·15 (1·11 to 1·2) | 1·07 (1·03 to 1·1) |
|  | North East | 1·1 (1·04 to 1·17) | 1·01 (0·96 to 1·06) |  | 1·08 (1·03 to 1·14) | 1·02 (0·97 to 1·06) |  | 1·13 (1·08 to 1·17) | 1·03 (0·99 to 1·06) |
|  | North West | 1·03 (0·99 to 1·09) | 1·05 (1·01 to 1·09) |  | 1·04 (0·99 to 1·08) | 1·03 (0·99 to 1·06) |  | 1·09 (1·05 to 1·13) | 1 (0·98 to 1·03) |
|  | South East | 1·1 (1·05 to 1·16) | 1·1 (1·06 to 1·14) |  | 1·14 (1·09 to 1·18) | 1·06 (1·03 to 1·1) |  | 1·12 (1·08 to 1·16) | 1·06 (1·03 to 1·09) |
|  | South West | 1·03 (0·98 to 1·09) | 1·05 (1 to 1·1) |  | 1·03 (0·98 to 1·08) | 0·99 (0·95 to 1·02) |  | 1·06 (1·02 to 1·1) | 1·01 (0·98 to 1·05) |
|  | West Midlands | 1·15 (1·1 to 1·21) | 1·14 (1·1 to 1·19) |  | 1·11 (1·06 to 1·16) | 1·11 (1·07 to 1·15) |  | 1·17 (1·12 to 1·21) | 1·08 (1·05 to 1·11) |
|  | Yorkshire Humber | 1·17 (1·11 to 1·23) | 1·13 (1·09 to 1·18) |  | 1·16 (1·11 to 1·22) | 1·09 (1·06 to 1·14) |  | 1·19 (1·14 to 1·23) | 1·11 (1·08 to 1·15) |
| **Health characteristics** | |  |  |  |  |  |  |  |  |
|  | Atrial fibrillation (QOF) | 1·18 (1·1 to 1·26) | 1·18 (1·13 to 1·23) |  | 1·24 (1·18 to 1·29) | 1·15 (1·11 to 1·18) |  | 1·18 (1·15 to 1·22) | 1·15 (1·13 to 1·18) |
|  | Asthma (QOF) | 0·78 (0·75 to 0·81) | 0·78 (0·75 to 0·81) |  | 0·8 (0·77 to 0·83) | 0·76 (0·74 to 0·79) |  | 0·8 (0·78 to 0·83) | 0·74 (0·71 to 0·76) |
|  | Cancer (QOF) | 2·95 (2·86 to 3·04) | 2·71 (2·64 to 2·79) |  | 2·45 (2·39 to 2·51) | 2·2 (2·15 to 2·24) |  | 2·08 (2·04 to 2·12) | 1·85 (1·82 to 1·88) |
|  | COPD (QOF) | 1·28 (1·22 to 1·33) | 1·31 (1·26 to 1·35) |  | 1·32 (1·27 to 1·36) | 1·22 (1·19 to 1·26) |  | 1·34 (1·31 to 1·38) | 1·3 (1·27 to 1·33) |
|  | Heart failure (QOF) | 1·71 (1·6 to 1·82) | 1·64 (1·56 to 1·71) |  | 1·57 (1·49 to 1·65) | 1·63 (1·57 to 1·69) |  | 1·61 (1·55 to 1·67) | 1·58 (1·54 to 1·62) |
|  | Palliative care (QOF) | 5·83 (5·58 to 6·09) | 4·7 (4·51 to 4·9) |  | 4·7 (4·52 to 4·88) | 3·9 (3·77 to 4·04) |  | 3·86 (3·74 to 3·97) | 3·62 (3·52 to 3·72) |
|  | Bipolar & schizophrenia (GPES) | 1·43 (1·33 to 1·53) | 1·44 (1·36 to 1·53) |  | 1·41 (1·33 to 1·51) | 1·4 (1·32 to 1·48) |  | 1·24 (1·17 to 1·3) | 1·26 (1·19 to 1·32) |
|  | Coronary heart disease (GPES) | 0·96 (0·91 to 1·01) | 0·96 (0·93 to 0·99) |  | 0·97 (0·94 to 1·01) | 0·95 (0·93 to 0·98) |  | 0·96 (0·93 to 0·98) | 0·97 (0·95 to 0·98) |
|  | Epilepsy (GPES) | 1·37 (1·28 to 1·46) | 1·44 (1·37 to 1·52) |  | 1·37 (1·29 to 1·45) | 1·23 (1·17 to 1·29) |  | 1·24 (1·18 to 1·3) | 1·27 (1·22 to 1·33) |
|  | Learning dis· & Downs (GPES) | 2·2 (2·01 to 2·4) | 2·16 (2·01 to 2·32) |  | 2·21 (2·02 to 2·41) | 1·99 (1·85 to 2·14) |  | 1·86 (1·71 to 2·03) | 1·61 (1·5 to 1·73) |
|  | Liver cirrhosis (GPES) | 2·6 (2·41 to 2·8) | 2·77 (2·62 to 2·92) |  | 2·19 (2·04 to 2·34) | 2·38 (2·26 to 2·51) |  | 2·08 (1·96 to 2·2) | 2·18 (2·07 to 2·29) |
|  | Stroke & TIA (GPES) | 1·14 (1·08 to 1·2) | 1·19 (1·15 to 1·24) |  | 1·22 (1·17 to 1·27) | 1·22 (1·18 to 1·25) |  | 1·17 (1·14 to 1·2) | 1·19 (1·16 to 1·21) |
|  | Chronic respiratory disease (HES) | 1·42 (1·36 to 1·47) | 1·38 (1·33 to 1·43) |  | 1·44 (1·4 to 1·49) | 1·42 (1·38 to 1·46) |  | 1·39 (1·35 to 1·42) | 1·42 (1·39 to 1·45) |
|  | Cardiovascular disease (HES) | 1·9 (1·85 to 1·96) | 1·74 (1·7 to 1·78) |  | 1·79 (1·75 to 1·84) | 1·6 (1·57 to 1·64) |  | 1·67 (1·64 to 1·7) | 1·54 (1·52 to 1·57) |
|  | Chronic kidney disease (QOF only) | 1·17 (1·1 to 1·25) | 1·24 (1·17 to 1·31) |  | 1·1 (1·06 to 1·15) | 1·12 (1·07 to 1·16) |  | 1·06 (1·03 to 1·09) | 1·09 (1·06 to 1·12) |
|  | Chronic kidney disease (HES) | 1·94 (1·85 to 2·04) | 1·88 (1·81 to 1·96) |  | 1·64 (1·58 to 1·7) | 1·71 (1·66 to 1·77) |  | 1·55 (1·51 to 1·59) | 1·58 (1·55 to 1·62) |
|  | Dementia (HES or QOF) | 2·77 (2·58 to 2·98) | 2·17 (2·04 to 2·31) |  | 2·42 (2·3 to 2·55) | 2·27 (2·18 to 2·38) |  | 2·87 (2·78 to 2·96) | 2·64 (2·57 to 2·72) |
|  | Diabetes (QOF only) | 1·29 (1·22 to 1·36) | 1·27 (1·22 to 1·32) |  | 1·25 (1·19 to 1·31) | 1·26 (1·22 to 1·3) |  | 1·29 (1·25 to 1·34) | 1·25 (1·22 to 1·29) |
|  | Diabetes (HES) | 1·4 (1·35 to 1·45) | 1·41 (1·37 to 1·45) |  | 1·41 (1·37 to 1·45) | 1·41 (1·38 to 1·44) |  | 1·37 (1·34 to 1·4) | 1·36 (1·33 to 1·38) |
|  | Clinically vulnerable (NIMS) | 1·87 (1·81 to 1·93) | 1·54 (1·5 to 1·58) |  | 1·63 (1·58 to 1·67) | 1·39 (1·36 to 1·43) |  | 1·35 (1·32 to 1·38) | 1·15 (1·13 to 1·17) |
|  | Care home resident (GPES) | 2·19 (1·99 to 2·41) | 2·27 (2·1 to 2·46) |  | 2·01 (1·87 to 2·16) | 1·93 (1·82 to 2·05) |  | 1·79 (1·71 to 1·87) | 1·81 (1·73 to 1·88) |
| **Smoking status** | |  |  |  |  |  |  |  |  |
|  | Never smoker (NHSD) ^ref^ |  |  |  |  |  |  |  |  |
|  | Current smoker (NHSD) | 2·2 (2·12 to 2·28) | 2·32 (2·25 to 2·38) |  | 2·26 (2·19 to 2·33) | 2·37 (2·31 to 2·43) |  | 2·27 (2·21 to 2·33) | 2·2 (2·15 to 2·25) |
|  | Ex-smoker (NHSD) | 1·34 (1·3 to 1·39) | 1·33 (1·3 to 1·36) |  | 1·33 (1·29 to 1·36) | 1·35 (1·32 to 1·38) |  | 1·33 (1·31 to 1·36) | 1·28 (1·26 to 1·31) |
|  | Non-smoker (NHSD) | 1·18 (1·09 to 1·28) | 1·16 (1·08 to 1·24) |  | 1·12 (1·04 to 1·2) | 1·22 (1·15 to 1·29) |  | 1·18 (1·12 to 1·24) | 1·2 (1·15 to 1·26) |
|  | No record (NHSD) | 2·26 (2·17 to 2·36) | 2·31 (2·23 to 2·39) |  | 2·26 (2·18 to 2·34) | 2·32 (2·25 to 2·39) |  | 2·35 (2·29 to 2·42) | 2·32 (2·27 to 2·38) |
| **Wave & COVID-19 & Vaccination status** | |  |  |  |  |  |  |  |  |
|  | Wave 1 & 0-4 weeks & unvaccinated | 64·44 (58·62 to 70·84) | 81·35 (75·87 to 87·22) |  | 77·62 (71·67 to 84·07) | 79·21 (74·62 to 84·07) |  | 57·07 (53·57 to 60·8) | 68·68 (65·49 to 72·03) |
|  | Wave 1 & 5-26 weeks & unvaccinated | 4·48 (3·81 to 5·28) | 5·72 (5·06 to 6·47) |  | 6·65 (5·85 to 7·55) | 6·01 (5·42 to 6·68) |  | 4·87 (4·39 to 5·4) | 4·92 (4·5 to 5·37) |
|  | Wave 2 & 0-4 weeks & unvaccinated | 28·23 (26·36 to 30·24) | 33·67 (31·93 to 35·5) |  | 39·15 (36·97 to 41·45) | 42·16 (40·29 to 44·12) |  | 41·05 (39·24 to 42·95) | 49·56 (47·78 to 51·41) |
|  | Wave 2 & 0-4 weeks & 1st vaccine | 6·94 (3·93 to 12·24) | 15·69 (10·81 to 22·77) |  | 19·57 (14·2 to 26·96) | 22·06 (17·02 to 28·6) |  | 22·85 (18·58 to 28·1) | 26·85 (22·75 to 31·69) |
|  | Wave 2 & 5-26 weeks & unvaccinated | 3·23 (2·96 to 3·52) | 3·91 (3·66 to 4·18) |  | 3·99 (3·69 to 4·3) | 4·34 (4·08 to 4·61) |  | 4·15 (3·9 to 4·41) | 4·86 (4·61 to 5·11) |
|  | Wave 2 & 5-26 weeks & 1st vaccine | 1·57 (0·93 to 2·66) | 2·83 (1·93 to 4·16) |  | 3·21 (2·26 to 4·57) | 3·02 (2·2 to 4·13) |  | 3·28 (2·58 to 4·17) | 4·14 (3·4 to 5·03) |
|  | Wave 3 & 0-4 weeks & unvaccinated | 38·6 (33·41 to 44·6) | 44·74 (39·67 to 50·47) |  | 41·87 (36·48 to 48·05) | 47·08 (41·87 to 52·93) |  | 42·05 (36·92 to 47·9) | 49·69 (44·48 to 55·52) |
|  | Wave 3 & 0-4 weeks & 1st vaccine | 20·96 (15·36 to 28·59) | 22·44 (17·48 to 28·8) |  | 22·03 (16·08 to 30·17) | 19·23 (14·53 to 25·47) |  | 20·62 (15·66 to 27·14) | 26·37 (21·11 to 32·93) |
|  | Wave 3 & 0-4 weeks & 2nd vaccine | 7·64 (6·79 to 8·6) | 8·2 (7·45 to 9·03) |  | 10·61 (9·62 to 11·7) | 10·62 (9·8 to 11·52) |  | 11·2 (10·32 to 12·17) | 13·18 (12·37 to 14·06) |
|  | Wave 3 & 0-4 weeks & booster vaccine | 2·48 (2·1 to 2·94) | 2·93 (2·57 to 3·35) |  | 3·41 (2·99 to 3·89) | 3·5 (3·15 to 3·89) |  | 4·18 (3·79 to 4·6) | 4·56 (4·23 to 4·93) |
|  | Wave 3 & 5-26 weeks & unvaccinated | 4·23 (3·49 to 5·12) | 5·88 (5·07 to 6·82) |  | 5·38 (4·53 to 6·4) | 4·96 (4·21 to 5·85) |  | 4·27 (3·55 to 5·15) | 5·18 (4·41 to 6·08) |
|  | Wave 3 & 5-26 weeks & 1st vaccine | 3·8 (2·78 to 5·21) | 3·71 (2·84 to 4·84) |  | 4·45 (3·27 to 6·04) | 4·76 (3·67 to 6·17) |  | 4·53 (3·49 to 5·88) | 4·14 (3·2 to 5·34) |
|  | Wave 3 & 5-26 weeks & 2nd vaccine | 1·59 (1·42 to 1·78) | 1·54 (1·4 to 1·7) |  | 2·04 (1·85 to 2·25) | 1·88 (1·73 to 2·04) |  | 2·1 (1·93 to 2·28) | 2·27 (2·12 to 2·42) |
|  | Wave 3 & 5-26 weeks & booster vaccine | 1·11 (0·99 to 1·24) | 1·33 (1·22 to 1·45) |  | 1·56 (1·43 to 1·71) | 1·57 (1·46 to 1·69) |  | 1·77 (1·66 to 1·9) | 1·95 (1·85 to 2·06) |
|  |  |  |  |  |  |  |  |  |  |
|  |  | **75-79 years** | |  | **80-84 years** | |  | **85-89 years** | |
|  |  | **Female** | **Male** |  | **Female** | **Male** |  | **Female** | **Male** |
| **Age** | | 1·09 (1·08 to 1·09) | 1·08 (1·08 to 1·09) |  | 1·1 (1·09 to 1·1) | 1·1 (1·1 to 1·1) |  | 1·11 (1·11 to 1·12) | 1·11 (1·11 to 1·12) |
| **Ethnicity** | |  |  |  |  |  |  |  |  |
|  | White ^ref^ |  |  |  |  |  |  |  |  |
|  | Black | 0·8 (0·75 to 0·85) | 0·83 (0·79 to 0·88) |  | 0·8 (0·77 to 0·84) | 0·94 (0·9 to 0·99) |  | 0·82 (0·78 to 0·87) | 0·91 (0·86 to 0·95) |
|  | Asian | 0·94 (0·91 to 0·98) | 0·87 (0·84 to 0·9) |  | 0·96 (0·93 to 1) | 0·91 (0·88 to 0·94) |  | 0·93 (0·89 to 0·96) | 0·98 (0·95 to 1·02) |
|  | Mixed | 0·85 (0·75 to 0·96) | 0·83 (0·74 to 0·93) |  | 0·89 (0·8 to 0·99) | 0·97 (0·87 to 1·07) |  | 0·94 (0·85 to 1·04) | 0·92 (0·82 to 1·03) |
|  | Other | 0·73 (0·64 to 0·83) | 0·72 (0·64 to 0·81) |  | 0·72 (0·63 to 0·81) | 0·8 (0·71 to 0·9) |  | 0·71 (0·63 to 0·8) | 0·8 (0·7 to 0·92) |
|  | Unknown | 1·59 (1·52 to 1·65) | 1·36 (1·31 to 1·41) |  | 1·49 (1·44 to 1·55) | 1·35 (1·3 to 1·4) |  | 1·36 (1·32 to 1·41) | 1·1 (1·05 to 1·15) |
| **IMD Quintile** | |  |  |  |  |  |  |  |  |
|  | Q5 (least deprived)  ^ref^ |  |  |  |  |  |  |  |  |
|  | Q4 | 1·06 (1·03 to 1·08) | 1·09 (1·06 to 1·11) |  | 1·07 (1·05 to 1·09) | 1·08 (1·06 to 1·09) |  | 1·06 (1·04 to 1·08) | 1·07 (1·05 to 1·09) |
|  | Q3 | 1·15 (1·12 to 1·17) | 1·17 (1·14 to 1·19) |  | 1·12 (1·1 to 1·14) | 1·14 (1·12 to 1·16) |  | 1·1 (1·08 to 1·12) | 1·13 (1·11 to 1·15) |
|  | Q2 | 1·21 (1·18 to 1·23) | 1·26 (1·24 to 1·29) |  | 1·2 (1·17 to 1·22) | 1·23 (1·21 to 1·26) |  | 1·16 (1·14 to 1·18) | 1·21 (1·19 to 1·23) |
|  | Q1 (most deprived) | 1·33 (1·3 to 1·36) | 1·37 (1·34 to 1·4) |  | 1·28 (1·25 to 1·3) | 1·35 (1·33 to 1·38) |  | 1·23 (1·21 to 1·26) | 1·3 (1·27 to 1·33) |
|  | Unknown | 5·03 (4·75 to 5·33) | 4·28 (4·07 to 4·51) |  | 4·15 (3·94 to 4·36) | 3·38 (3·22 to 3·55) |  | 3·37 (3·22 to 3·54) | 2·81 (2·67 to 2·96) |
| **Region** | |  |  |  |  |  |  |  |  |
|  | London ^ref^ |  |  |  |  |  |  |  |  |
|  | East Midlands | 1·1 (1·06 to 1·13) | 1·1 (1·07 to 1·13) |  | 1·1 (1·07 to 1·13) | 1·09 (1·07 to 1·13) |  | 1·12 (1·09 to 1·15) | 1·05 (1·02 to 1·08) |
|  | East of England | 1·13 (1·09 to 1·17) | 1·06 (1·03 to 1·09) |  | 1·12 (1·09 to 1·15) | 1·09 (1·06 to 1·12) |  | 1·15 (1·12 to 1·18) | 1·07 (1·04 to 1·1) |
|  | North East | 1·06 (1·02 to 1·1) | 1·06 (1·02 to 1·1) |  | 1·08 (1·05 to 1·12) | 1·07 (1·03 to 1·1) |  | 1·07 (1·04 to 1·1) | 1·02 (0·99 to 1·06) |
|  | North West | 1·03 (1 to 1·06) | 1·02 (0·99 to 1·05) |  | 1·01 (0·99 to 1·04) | 1·02 (1 to 1·05) |  | 1·03 (1·01 to 1·06) | 0·99 (0·97 to 1·02) |
|  | South East | 1·06 (1·03 to 1·09) | 1·06 (1·04 to 1·09) |  | 1·05 (1·02 to 1·07) | 1·07 (1·05 to 1·1) |  | 1·05 (1·03 to 1·08) | 1·06 (1·03 to 1·09) |
|  | South West | 1·05 (1·01 to 1·08) | 1 (0·97 to 1·03) |  | 1·07 (1·04 to 1·1) | 1·04 (1·01 to 1·06) |  | 1·06 (1·04 to 1·09) | 1·02 (0·99 to 1·05) |
|  | West Midlands | 1·1 (1·06 to 1·13) | 1·08 (1·05 to 1·11) |  | 1·06 (1·04 to 1·09) | 1·06 (1·03 to 1·09) |  | 1·05 (1·02 to 1·07) | 1·03 (1 to 1·06) |
|  | Yorkshire Humber | 1·16 (1·13 to 1·2) | 1·11 (1·08 to 1·14) |  | 1·14 (1·11 to 1·17) | 1·12 (1·09 to 1·15) |  | 1·15 (1·12 to 1·18) | 1·08 (1·05 to 1·11) |
| **Health characteristics** | |  |  |  |  |  |  |  |  |
|  | Atrial fibrillation (QOF) | 1·23 (1·2 to 1·26) | 1·15 (1·13 to 1·17) |  | 1·23 (1·21 to 1·25) | 1·13 (1·11 to 1·15) |  | 1·24 (1·22 to 1·25) | 1·16 (1·14 to 1·17) |
|  | Asthma (QOF) | 0·8 (0·78 to 0·82) | 0·78 (0·76 to 0·8) |  | 0·83 (0·81 to 0·85) | 0·83 (0·81 to 0·85) |  | 0·87 (0·85 to 0·89) | 0·84 (0·82 to 0·87) |
|  | Cancer (QOF) | 1·78 (1·75 to 1·82) | 1·55 (1·53 to 1·57) |  | 1·54 (1·52 to 1·57) | 1·41 (1·39 to 1·43) |  | 1·34 (1·32 to 1·36) | 1·29 (1·27 to 1·31) |
|  | COPD (QOF) | 1·43 (1·4 to 1·47) | 1·34 (1·32 to 1·37) |  | 1·43 (1·4 to 1·46) | 1·36 (1·33 to 1·38) |  | 1·39 (1·36 to 1·42) | 1·28 (1·25 to 1·31) |
|  | Heart failure (QOF) | 1·58 (1·53 to 1·62) | 1·52 (1·49 to 1·56) |  | 1·52 (1·49 to 1·56) | 1·56 (1·53 to 1·59) |  | 1·45 (1·42 to 1·48) | 1·54 (1·51 to 1·57) |
|  | Palliative care (QOF) | 3·01 (2·94 to 3·09) | 3·01 (2·94 to 3·08) |  | 2·37 (2·32 to 2·42) | 2·37 (2·32 to 2·42) |  | 1·9 (1·87 to 1·94) | 1·93 (1·89 to 1·98) |
|  | Bipolar & schizophrenia (GPES) | 1·16 (1·11 to 1·22) | 1·24 (1·18 to 1·31) |  | 1·29 (1·23 to 1·35) | 1·2 (1·13 to 1·28) |  | 1·26 (1·2 to 1·34) | 1·23 (1·13 to 1·33) |
|  | Coronary heart disease (GPES) | 0·99 (0·97 to 1·01) | 0·99 (0·97 to 1) |  | 1·01 (0·99 to 1·02) | 0·99 (0·98 to 1) |  | 1·05 (1·04 to 1·07) | 1·03 (1·01 to 1·04) |
|  | Epilepsy (GPES) | 1·18 (1·13 to 1·23) | 1·17 (1·12 to 1·21) |  | 1·18 (1·13 to 1·23) | 1·24 (1·19 to 1·29) |  | 1·19 (1·14 to 1·24) | 1·17 (1·12 to 1·22) |
|  | Learning dis· & Downs (GPES) | 1·61 (1·46 to 1·77) | 1·56 (1·43 to 1·7) |  | 1·49 (1·32 to 1·67) | 1·62 (1·45 to 1·81) |  | 1·61 (1·38 to 1·88) | 1·33 (1·13 to 1·56) |
|  | Liver cirrhosis (GPES) | 1·91 (1·79 to 2·02) | 2·02 (1·91 to 2·14) |  | 1·86 (1·75 to 1·99) | 1·87 (1·75 to 2) |  | 1·64 (1·52 to 1·78) | 1·59 (1·45 to 1·74) |
|  | Stroke & TIA (GPES) | 1·17 (1·15 to 1·2) | 1·14 (1·12 to 1·16) |  | 1·17 (1·15 to 1·19) | 1·16 (1·14 to 1·18) |  | 1·16 (1·14 to 1·18) | 1·15 (1·13 to 1·17) |
|  | Chronic respiratory disease (HES) | 1·38 (1·35 to 1·41) | 1·4 (1·38 to 1·43) |  | 1·33 (1·31 to 1·36) | 1·37 (1·35 to 1·4) |  | 1·27 (1·24 to 1·29) | 1·32 (1·29 to 1·34) |
|  | Cardiovascular disease (HES) | 1·56 (1·53 to 1·58) | 1·48 (1·46 to 1·51) |  | 1·44 (1·42 to 1·46) | 1·43 (1·41 to 1·45) |  | 1·37 (1·36 to 1·39) | 1·42 (1·39 to 1·44) |
|  | Chronic kidney disease (QOF only) | 1·05 (1·03 to 1·07) | 1·08 (1·05 to 1·1) |  | 1·05 (1·03 to 1·07) | 1·05 (1·03 to 1·07) |  | 1·05 (1·03 to 1·06) | 1·07 (1·05 to 1·09) |
|  | Chronic kidney disease (HES) | 1·47 (1·44 to 1·5) | 1·54 (1·51 to 1·57) |  | 1·42 (1·39 to 1·44) | 1·48 (1·45 to 1·5) |  | 1·36 (1·34 to 1·38) | 1·41 (1·39 to 1·43) |
|  | Dementia (HES or QOF) | 2·97 (2·9 to 3·03) | 2·8 (2·74 to 2·86) |  | 2·95 (2·9 to 3) | 2·83 (2·79 to 2·88) |  | 2·92 (2·87 to 2·96) | 2·55 (2·51 to 2·6) |
|  | Diabetes (QOF only) | 1·26 (1·22 to 1·3) | 1·23 (1·2 to 1·26) |  | 1·17 (1·14 to 1·21) | 1·2 (1·17 to 1·23) |  | 1·15 (1·12 to 1·19) | 1·17 (1·13 to 1·2) |
|  | Diabetes (HES) | 1·31 (1·29 to 1·34) | 1·32 (1·29 to 1·34) |  | 1·32 (1·3 to 1·34) | 1·3 (1·28 to 1·32) |  | 1·26 (1·24 to 1·28) | 1·24 (1·22 to 1·26) |
|  | Clinically vulnerable (NIMS) | 0·96 (0·94 to 0·98) | 0·82 (0·81 to 0·84) |  | 0·66 (0·65 to 0·67) | 0·54 (0·54 to 0·55) |  | 0·46 (0·46 to 0·47) | 0·41 (0·4 to 0·41) |
|  | Care home resident (GPES) | 1·72 (1·67 to 1·78) | 1·67 (1·62 to 1·72) |  | 1·67 (1·63 to 1·71) | 1·7 (1·66 to 1·75) |  | 1·59 (1·56 to 1·62) | 1·6 (1·57 to 1·64) |
| **Smoking status** | |  |  |  |  |  |  |  |  |
|  | Never smoker (NHSD) ^ref^ |  |  |  |  |  |  |  |  |
|  | Current smoker (NHSD) | 2·05 (2 to 2·1) | 1·93 (1·89 to 1·98) |  | 1·79 (1·75 to 1·84) | 1·71 (1·66 to 1·75) |  | 1·6 (1·55 to 1·64) | 1·5 (1·45 to 1·55) |
|  | Ex-smoker (NHSD) | 1·26 (1·23 to 1·28) | 1·22 (1·2 to 1·24) |  | 1·2 (1·18 to 1·21) | 1·16 (1·14 to 1·17) |  | 1·1 (1·09 to 1·12) | 1·1 (1·09 to 1·12) |
|  | Non-smoker (NHSD) | 1·19 (1·13 to 1·24) | 1·2 (1·15 to 1·25) |  | 1·14 (1·1 to 1·18) | 1·12 (1·07 to 1·16) |  | 1·1 (1·07 to 1·14) | 1·1 (1·06 to 1·14) |
|  | No record (NHSD) | 2·44 (2·38 to 2·49) | 2·35 (2·3 to 2·4) |  | 2·49 (2·44 to 2·54) | 2·55 (2·5 to 2·6) |  | 2·58 (2·53 to 2·62) | 2·63 (2·58 to 2·68) |
| **Wave & COVID-19 & Vaccination status** | |  |  |  |  |  |  |  |  |
|  | Wave 1 & 0-4 weeks & unvaccinated | 43·67 (41·48 to 45·98) | 52·66 (50·53 to 54·88) |  | 30·22 (28·98 to 31·52) | 35·06 (33·81 to 36·37) |  | 18·22 (17·53 to 18·95) | 22·54 (21·71 to 23·39) |
|  | Wave 1 & 5-26 weeks & unvaccinated | 3·82 (3·5 to 4·17) | 4·79 (4·44 to 5·16) |  | 3·44 (3·22 to 3·68) | 4·18 (3·92 to 4·46) |  | 2·98 (2·81 to 3·15) | 3·22 (3·01 to 3·44) |
|  | Wave 2 & 0-4 weeks & unvaccinated | 38·34 (36·89 to 39·85) | 49·08 (47·52 to 50·7) |  | 30·78 (29·79 to 31·8) | 41·43 (40·21 to 42·68) |  | 21·38 (20·75 to 22·03) | 28·42 (27·56 to 29·31) |
|  | Wave 2 & 0-4 weeks & 1st vaccine | 21·39 (18·52 to 24·7) | 30 (26·66 to 33·75) |  | 19·67 (17·82 to 21·72) | 27·66 (25·39 to 30·14) |  | 15·46 (14·15 to 16·88) | 22·53 (20·69 to 24·54) |
|  | Wave 2 & 5-26 weeks & unvaccinated | 4·17 (3·96 to 4·39) | 4·53 (4·32 to 4·75) |  | 3·96 (3·79 to 4·13) | 4·24 (4·05 to 4·44) |  | 3·36 (3·23 to 3·49) | 4·36 (4·17 to 4·56) |
|  | Wave 2 & 5-26 weeks & 1st vaccine | 2·9 (2·43 to 3·46) | 3·13 (2·64 to 3·71) |  | 3·21 (2·85 to 3·6) | 3·96 (3·54 to 4·43) |  | 2·53 (2·27 to 2·82) | 3·39 (3·01 to 3·81) |
|  | Wave 3 & 0-4 weeks & unvaccinated | 39·62 (35·21 to 44·58) | 45·8 (40·71 to 51·52) |  | 33·45 (29·88 to 37·45) | 42·72 (38·38 to 47·53) |  | 24·25 (21·52 to 27·32) | 35·39 (31·02 to 40·37) |
|  | Wave 3 & 0-4 weeks & 1st vaccine | 20·42 (15·98 to 26·1) | 19·82 (15·59 to 25·19) |  | 21·7 (17·85 to 26·38) | 22·3 (18·38 to 27·06) |  | 13·79 (11·26 to 16·88) | 21·11 (17·17 to 25·97) |
|  | Wave 3 & 0-4 weeks & 2nd vaccine | 13·39 (12·46 to 14·4) | 16·54 (15·62 to 17·52) |  | 13·62 (12·77 to 14·52) | 18·06 (17·1 to 19·07) |  | 11·57 (10·88 to 12·32) | 16·48 (15·55 to 17·46) |
|  | Wave 3 & 0-4 weeks & booster vaccine | 5·01 (4·63 to 5·43) | 5·94 (5·57 to 6·33) |  | 5·36 (5·02 to 5·71) | 7·3 (6·92 to 7·71) |  | 5·06 (4·77 to 5·36) | 7·41 (7·01 to 7·84) |
|  | Wave 3 & 5-26 weeks & unvaccinated | 3·63 (3·02 to 4·36) | 4·89 (4·1 to 5·84) |  | 4·39 (3·78 to 5·1) | 4·09 (3·43 to 4·88) |  | 3·65 (3·13 to 4·25) | 3·86 (3·12 to 4·78) |
|  | Wave 3 & 5-26 weeks & 1st vaccine | 4·66 (3·69 to 5·88) | 4·03 (3·16 to 5·14) |  | 4·03 (3·25 to 5) | 3·75 (2·98 to 4·72) |  | 3·08 (2·51 to 3·78) | 3·42 (2·65 to 4·43) |
|  | Wave 3 & 5-26 weeks & 2nd vaccine | 2·58 (2·4 to 2·77) | 2·47 (2·31 to 2·64) |  | 2·94 (2·76 to 3·13) | 2·77 (2·6 to 2·95) |  | 2·59 (2·44 to 2·75) | 3·06 (2·87 to 3·26) |
|  | Wave 3 & 5-26 weeks & booster vaccine | 2·19 (2·07 to 2·31) | 2·3 (2·19 to 2·41) |  | 2·38 (2·28 to 2·49) | 2·61 (2·5 to 2·72) |  | 2·37 (2·27 to 2·47) | 2·93 (2·8 to 3·06) |
|  |  |  |  |  |  |  |  |  |  |
|  |  | **90+ years** | |  |  | |  |  | |
|  |  | **Female** | **Male** |  |  |  |  |  |  |
| **Age** | | 1·07 (1·07 to 1·07) | 1·08 (1·08 to 1·08) |  |  |  |  |  |  |
| **Ethnicity** | |  |  |  |  |  |  |  |  |
|  | White ^ref^ |  |  |  |  |  |  |  |  |
|  | Black | 0·87 (0·83 to 0·92) | 0·89 (0·83 to 0·94) |  |  |  |  |  |  |
|  | Asian | 0·84 (0·81 to 0·88) | 0·8 (0·76 to 0·84) |  |  |  |  |  |  |
|  | Mixed | 0·9 (0·82 to 1) | 0·95 (0·83 to 1·09) |  |  |  |  |  |  |
|  | Other | 0·81 (0·73 to 0·9) | 0·77 (0·66 to 0·89) |  |  |  |  |  |  |
|  | Unknown | 0·9 (0·87 to 0·93) | 0·41 (0·39 to 0·43) |  |  |  |  |  |  |
| **IMD Quintile** | |  |  |  |  |  |  |  |  |
|  | Q5 (least deprived)  ^ref^ |  |  |  |  |  |  |  |  |
|  | Q4 | 1·07 (1·06 to 1·09) | 1·08 (1·06 to 1·1) |  |  |  |  |  |  |
|  | Q3 | 1·11 (1·09 to 1·12) | 1·14 (1·12 to 1·16) |  |  |  |  |  |  |
|  | Q2 | 1·14 (1·12 to 1·16) | 1·18 (1·15 to 1·21) |  |  |  |  |  |  |
|  | Q1 (most deprived) | 1·17 (1·15 to 1·19) | 1·23 (1·2 to 1·26) |  |  |  |  |  |  |
|  | Unknown | 2·31 (2·21 to 2·41) | 2·15 (2·02 to 2·28) |  |  |  |  |  |  |
| **Region** | |  |  |  |  |  |  |  |  |
|  | London ^ref^ |  |  |  |  |  |  |  |  |
|  | East Midlands | 1·1 (1·07 to 1·13) | 1·07 (1·04 to 1·11) |  |  |  |  |  |  |
|  | East of England | 1·16 (1·13 to 1·18) | 1·1 (1·06 to 1·13) |  |  |  |  |  |  |
|  | North East | 1·13 (1·1 to 1·16) | 1·03 (0·99 to 1·07) |  |  |  |  |  |  |
|  | North West | 1·04 (1·02 to 1·06) | 1 (0·97 to 1·04) |  |  |  |  |  |  |
|  | South East | 1·08 (1·06 to 1·11) | 1·09 (1·06 to 1·12) |  |  |  |  |  |  |
|  | South West | 1·11 (1·08 to 1·13) | 1·06 (1·03 to 1·09) |  |  |  |  |  |  |
|  | West Midlands | 1·05 (1·03 to 1·07) | 1·04 (1 to 1·07) |  |  |  |  |  |  |
|  | Yorkshire Humber | 1·17 (1·14 to 1·2) | 1·09 (1·06 to 1·13) |  |  |  |  |  |  |
| **Health characteristics** | |  |  |  |  |  |  |  |  |
|  | Atrial fibrillation (QOF) | 1·21 (1·19 to 1·22) | 1·16 (1·14 to 1·18) |  |  |  |  |  |  |
|  | Asthma (QOF) | 0·89 (0·86 to 0·91) | 0·89 (0·86 to 0·92) |  |  |  |  |  |  |
|  | Cancer (QOF) | 1·17 (1·15 to 1·18) | 1·18 (1·16 to 1·2) |  |  |  |  |  |  |
|  | COPD (QOF) | 1·3 (1·27 to 1·33) | 1·23 (1·19 to 1·26) |  |  |  |  |  |  |
|  | Heart failure (QOF) | 1·38 (1·36 to 1·4) | 1·41 (1·38 to 1·44) |  |  |  |  |  |  |
|  | Palliative care (QOF) | 1·49 (1·47 to 1·51) | 1·56 (1·53 to 1·6) |  |  |  |  |  |  |
|  | Bipolar & schizophrenia (GPES) | 1·25 (1·18 to 1·33) | 1·01 (0·89 to 1·14) |  |  |  |  |  |  |
|  | Coronary heart disease (GPES) | 1·05 (1·04 to 1·07) | 1·04 (1·02 to 1·05) |  |  |  |  |  |  |
|  | Epilepsy (GPES) | 1·18 (1·13 to 1·24) | 1·15 (1·09 to 1·22) |  |  |  |  |  |  |
|  | Learning dis· & Downs (GPES) | 1·41 (1·14 to 1·73) | 1·11 (0·84 to 1·46) |  |  |  |  |  |  |
|  | Liver cirrhosis (GPES) | 1·46 (1·31 to 1·62) | 1·35 (1·15 to 1·59) |  |  |  |  |  |  |
|  | Stroke & TIA (GPES) | 1·15 (1·13 to 1·16) | 1·13 (1·11 to 1·16) |  |  |  |  |  |  |
|  | Chronic respiratory disease (HES) | 1·18 (1·16 to 1·2) | 1·23 (1·2 to 1·26) |  |  |  |  |  |  |
|  | Cardiovascular disease (HES) | 1·32 (1·3 to 1·34) | 1·41 (1·38 to 1·44) |  |  |  |  |  |  |
|  | Chronic kidney disease (QOF only) | 1·07 (1·05 to 1·08) | 1·12 (1·1 to 1·14) |  |  |  |  |  |  |
|  | Chronic kidney disease (HES) | 1·27 (1·25 to 1·28) | 1·32 (1·29 to 1·34) |  |  |  |  |  |  |
|  | Dementia (HES or QOF) | 2·44 (2·41 to 2·47) | 1·99 (1·95 to 2·02) |  |  |  |  |  |  |
|  | Diabetes (QOF only) | 1·09 (1·06 to 1·12) | 1·11 (1·07 to 1·15) |  |  |  |  |  |  |
|  | Diabetes (HES) | 1·16 (1·15 to 1·18) | 1·17 (1·14 to 1·19) |  |  |  |  |  |  |
|  | Clinically vulnerable (NIMS) | 0·37 (0·36 to 0·37) | 0·29 (0·28 to 0·29) |  |  |  |  |  |  |
|  | Care home resident (GPES) | 1·41 (1·39 to 1·43) | 1·36 (1·32 to 1·39) |  |  |  |  |  |  |
| **Smoking status** | |  |  |  |  |  |  |  |  |
|  | Never smoker (NHSD) ^ref^ |  |  |  |  |  |  |  |  |
|  | Current smoker (NHSD) | 1·32 (1·27 to 1·37) | 1·31 (1·25 to 1·38) |  |  |  |  |  |  |
|  | Ex-smoker (NHSD) | 1·05 (1·04 to 1·06) | 1·07 (1·05 to 1·08) |  |  |  |  |  |  |
|  | Non-smoker (NHSD) | 1·05 (1·02 to 1·08) | 1·07 (1·02 to 1·11) |  |  |  |  |  |  |
|  | No record (NHSD) | 2·35 (2·31 to 2·39) | 2·15 (2·1 to 2·2) |  |  |  |  |  |  |
| **Wave & COVID-19 & Vaccination status** | |  |  |  |  |  |  |  |  |
|  | Wave 1 & 0-4 weeks & unvaccinated | 11·44 (11·05 to 11·85) | 14·57 (13·97 to 15·19) |  |  |  |  |  |  |
|  | Wave 1 & 5-26 weeks & unvaccinated | 2·23 (2·12 to 2·35) | 2·71 (2·52 to 2·91) |  |  |  |  |  |  |
|  | Wave 2 & 0-4 weeks & unvaccinated | 13·96 (13·59 to 14·35) | 18·01 (17·38 to 18·66) |  |  |  |  |  |  |
|  | Wave 2 & 0-4 weeks & 1st vaccine | 10·26 (9·48 to 11·1) | 17·2 (15·66 to 18·9) |  |  |  |  |  |  |
|  | Wave 2 & 5-26 weeks & unvaccinated | 2·51 (2·43 to 2·6) | 3·38 (3·2 to 3·57) |  |  |  |  |  |  |
|  | Wave 2 & 5-26 weeks & 1st vaccine | 2·21 (2·02 to 2·42) | 3·44 (3·03 to 3·92) |  |  |  |  |  |  |
|  | Wave 3 & 0-4 weeks & unvaccinated | 18·35 (16·23 to 20·74) | 25·87 (21·86 to 30·62) |  |  |  |  |  |  |
|  | Wave 3 & 0-4 weeks & 1st vaccine | 7·58 (5·96 to 9·63) | 17·21 (13·33 to 22·22) |  |  |  |  |  |  |
|  | Wave 3 & 0-4 weeks & 2nd vaccine | 8·41 (7·89 to 8·97) | 14·94 (13·92 to 16·04) |  |  |  |  |  |  |
|  | Wave 3 & 0-4 weeks & booster vaccine | 3·8 (3·59 to 4·02) | 6·57 (6·15 to 7·02) |  |  |  |  |  |  |
|  | Wave 3 & 5-26 weeks & unvaccinated | 2·7 (2·27 to 3·22) | 3·19 (2·43 to 4·19) |  |  |  |  |  |  |
|  | Wave 3 & 5-26 weeks & 1st vaccine | 2·53 (2·06 to 3·09) | 3·09 (2·26 to 4·23) |  |  |  |  |  |  |
|  | Wave 3 & 5-26 weeks & 2nd vaccine | 2·19 (2·07 to 2·33) | 2·81 (2·58 to 3·05) |  |  |  |  |  |  |
|  | Wave 3 & 5-26 weeks & booster vaccine | 1·95 (1·87 to 2·03) | 2·6 (2·46 to 2·75) |  |  |  |  |  |  |
